# AAV Vector Toolkit for the Delivery and Expression of the Artificial microRNA in the Murine Heart

**DOI:** 10.3390/biotech15030055

**Published:** 2026-07-17

**Authors:** Ivan I. Galkin, Viktoriia V. Skopenkova, Maria Y. Shubina, Anna V. Polikarpova, Svetlana G. Vassilieva, Irina M. Savchenko, Olga S. Lebedeva, Daria V. Goliusova, Margarita Y. Sharikova, Vladimir V. Gureev, Tatiana N. Malorodova, Alexey V. Deikin, Tatiana V. Egorova, Maryana V. Bardina

**Affiliations:** 1 Laboratory of Modeling and Gene Therapy of Hereditary Diseases, Institute of Gene Biology, Russian Academy of Sciences, Moscow 119334, Russia; i.galkin.marlin@gmail.com (I.I.G.);; 2 Laboratory of Genetic Technologies and Genome Editing for Biomedicine and Animal Health, Joint Center for Genetic Technologies, Belgorod National Research University, Belgorod 308015, Russia; 3 Marlin Biotech, Sochi 354340, Russia; 4 Lopukhin Federal Research and Clinical Center of Physical-Chemical Medicine of Federal Medical Biological Agency, Moscow 119435, Russia

**Keywords:** adeno-associated virus vectors, cardiac diseases, transgene delivery, cardiac promoters, myotropic serotypes, gene therapy

## Abstract

Background: Adeno-associated virus (AAV) vector-mediated gene transfer is an emerging treatment strategy for severe cardiac disorders with genetic etiology. We refined the AAV toolkit to achieve efficient and selective expression of the therapeutic transgene in mouse hearts. Methods: Using vectors with a reporter transgene, we evaluated AAV administration routes, AAV serotype tropism to the myocardium, and cardiospecific promoters. Results: We showed that systemic AAV administration provides potent delivery and uniform transduction of cardiac tissue, outperforming localized injection techniques. The MyoAAV 2A capsid variant enabled an improved heart-to-liver transduction ratio compared to the parental AAV9 serotype. Screening a panel of cardiac and pan-muscular promoters in vitro and in vivo verified the superiority of the cardiac troponin T (cTnT) promoter for robust heart-specific transgene expression. Finally, we demonstrated that the cumulative properties of systemic AAV delivery, the MyoAAV 2A serotype, and the cTnT promoter allowed for efficient cardiac synthesis of the therapeutic transgene—an artificial miRNA designed for the gene suppression strategy of FLNC-related cardiomyopathy. Conclusions: Our findings establish an effective AAV approach for transgene transfer into the mouse heart and promote the development of gene therapy for cardiac disorders.

## 1. Introduction

Cardiac diseases are the leading causes of death in the human population, with the number of cardiovascular disease-related deaths increasing from 13.1 million in 1990 to 19.2 million in 2023 [1]. An emerging treatment of inherited heart conditions is gene therapy [2], which utilizes adeno-associated virus (AAV) vectors as a delivery system [3,4]. Depending on the underlying molecular cause, the therapeutic approach is aimed at gene replacement [5,6,7], gene addition [8,9,10], gene suppression [11,12,13,14], or genome editing [15,16,17]. While AAV therapies based on gene augmentation have already entered clinical trials (NCT04179643; NCT05836259; NCT06092034; NCT06061549) gene suppression and genome editing therapies largely remain under preclinical development.

One of the challenges in developing gene therapy for cardiac disorders is achieving safe and efficient delivery of therapeutic genes (transgenes) to the hearts, minimizing vector distribution and transgene expression in non-target organs. The tissue tropism of AAV is provided by capsid surface motifs, which are determined by serotype. Naturally occurring AAV serotypes demonstrate robust affinity for skeletal and cardiac muscles but preferentially accumulate in the liver and transduce kidney, lung, central nervous system, and other tissues [18]. The AAV serotype 9 (AAV9) is the current gold standard for muscle gene transfer in preclinical studies [7,12,15] and gene therapy in humans (NCT06228924, NCT05793307, NCT03362502, NCT03368742, etc.). AAV9 hepatotoxicity [19] and the need for cardiac-specific transduction have led to engineering, by directed evolution or rational design, numerous AAV9-based capsid variants with improved characteristics [20,21,22,23].

The vector biodistribution and specificity of therapeutic transgenes expression are influenced by the mode of AAV administration [24]. Various delivery approaches ranging from systemic administration to local heart injections have been tested in animal models. In rodents, the most accessible methods of cardiac AAV delivery are intravenous injections via the tail vein or retro-orbital sinus [7,25]. In large animals, the size of the heart allows for local administration methods such as intracoronary infusions, intramyocardial injections through a surgical or catheter-mediated approach, and intrapericardial injection [24]. Local administration allows reducing the vector dose per animal and preventing the off-target activity, including causing an immune response. Despite the advantages of heart-targeted injections, the intravenous administration of AAV is preferably used in clinical trials due to its minimally invasive nature (NCT06228924, NCT06092034, NCT05793307, NCT05445323, etc.).

To further improve cardiac specificity, the tissue-targeted promoters are used to drive expression of the therapeutic transgene. Constitutive promoters such as the hybrid chicken beta-actin promoter with CMV enhancer (CAG) [26] provide robust transgene expression in muscles but lack tissue specificity. Muscle-specific promoters such as MHCK7, CK8, and SPc5-12 are used in AAV-based gene therapies for muscular disorders [27,28]; however, these promoters do not have a strong preference for myocardium over skeletal muscles. Several promoters have been proposed as heart-specific [29,30], but many of them still lack preclinical data on their safety. Therefore, the development of an optimal heart-restricted promoter remains an area of active research.

In the study, we revised AAV delivery methodology and vector design to achieve cardiac-specific transgene delivery and expression in mouse hearts. Using vectors with the reporter, we systematically evaluated three key components of the AAV toolkit: the AAV administration route, the AAV serotype, and the promoter in the expression cassette. Ultimately, we identified the best combination of the selected AAV elements and applied this combination to introduce a transgene with therapeutic potential into mouse hearts. As a transgene, we used an artificial microRNA; this type of non-coding transgene is frequently utilized in gene suppression approaches to silence faulty genes in an allele-selective manner via the cellular RNA interference (RNAi) pathway [11,31,32].

## 2. Materials and Methods

### 2.1. Ethics Statement

The experimental procedures involving mice were conducted in strict compliance with the European Communities Council Directive 2010/63/EU for animal experiments. The procedures were thoroughly reviewed and approved by the local Ethics Committee of the Institute of Gene Biology (protocol codes nos. 44 of 15 February 2022 and 45 of 15 June 2022) and Belgorod National Research University (protocol codes nos. 01-03и/22 of 29 March 2022 and 01-08и/23 of 07 August 2023). Cell experiments involving a patient with restrictive cardiomyopathy were conducted in accordance with the Declaration of Helsinki and were approved by the Ethical Board of Lopukhin Federal Research and Clinical Center of Physical-Chemical Medicine (protocol code no. 1 of 1 June 2021). The informed consent for the study was obtained from the patient’s parents.

### 2.2. Animals

C57BL/10ScSnJ (B10), C57BL/10ScSn-Dmdmdx (mdx), and C57BL/6 (B6) mouse strains were procured from the mouse bank “Pushchino” (Russia) and maintained under standard conditions. Briefly, 8–12-week-old mice were housed on a 12-h light/dark cycle with unrestricted access to food and water. Animals were randomized into treatment groups, and analyses were performed by operators blinded to the treatment status.

### 2.3. Cell Culture

All cells were cultured in standard conditions (5% CO_2_, 37 °C, humidity 80%). Adherent HEK293T cells, HepG2 cells, and HeLa cells (ATCC, Manassas, VA, USA) were maintained in DMEM (Gibco, Paisley, UK) supplemented with 10% fetal bovine serum (BioSera, Cholet, France), 1 mM sodium pyruvate (Paneco, Moscow, Russia), and 50 U/mL of penicillin/streptomycin (PanEco). MB135 cells were provided by Stephen Tapscott [33]. The cells were maintained in a mix of DMEM (Gibco) with Medium 199 with glutamine and Earle’s Salts (C210E, Paneco, Moscow, Russia) in a 4:1 proportion, supplemented with 15% fetal bovine serum, 50 U/mL of penicillin/streptomycin, 10 ng/mL of dexamethasone, and 10 ng/mL of human FGF-2 (07050, PanEco).

A skin biopsy was obtained from a young male patient with restrictive cardiomyopathy, caused by a de novo in-frame mutation in *FLNC* (c.7416_7418delGAA p.Glu2472_Asn2473delinsAsp) [34]. Consent for the study was obtained from the patient’s parents. The protocol was approved by the Ethical Board of Lopukhin Federal Research and Clinical Center of Physical-Chemical Medicine (protocol code no. 1 of 01.06.2021). The patient-specific iPSC line (IPSFIL14S) was obtained from skin fibroblasts by a non-integrating reprogramming method and characterized as previously described [35] (see Supplementary Information File). The healthy control iPSC line (iPS12) was previously described [36]. The iPSCs were maintained under standard conditions on the hESC-qualified Matrigel-covered (Corning, Bedford, MA, USA) 35 mm Petri dishes in mTeSR1 (Stemcell Technologies, Vancouver, BC, Canada) supplemented with 50 U/mL of penicillin/streptomycin (PanEco). For subculturing, iPSCs were detached by treatment with 0.5% trypsin-EDTA (Gibco, Grand Island, NY, USA) and replated at a density of 20,000 cells per cm^2^ in mTeSR1 supplemented with 10 μM ROCK-kinase inhibitor Y-27632 (DC Chemicals, Shanghai, China). The following daily medium exchanges were performed with mTeSR1 without Y-27632.

Differentiation of iPSCs into cardiomyocytes was performed in Matrigel-coated 24-well culture plates with the STEMdiff Ventricular Cardiomyocyte Differentiation Kit (Stemcell Technologies, Vancouver, BC, Canada) according to the manufacturer’s protocol. Spontaneous contractions were observed 8–10 days post-differentiation start. On days 10–14, to increase the purity of the cardiomyocytes, differentiated cells were exposed to selective conditions in glucose-free lactate-containing CDM3L medium: RPMI-1640 w/o glucose (Gibco, Grand Island, NY, USA), 0.5 mg/mL recombinant human albumin (eEnzyme, Gaithersburg, MD, USA), 1.2 mM ascorbic acid (Sigma-Aldrich, St. Louis, MO, USA), 4 mM sodium lactate (Panreac, Barcelona, Spain), and 1x GlutaMAX (Gibco, Grand Island, NY, USA). The iPSC-derived cardiomyocytes were cultured in STEMdiff Cardiomyocyte Maintenance Medium (Stemcell Technologies, Vancouver, BC, Canada); the medium was replenished every 2 days. For subculturing, the cardiomyocytes were detached with 0.25% trypsin-EDTA and reseeded at a ratio of 1:2 or 1:4 in 24-well or 4-well culture plates in STEMdiff Cardiomyocyte Maintenance Medium with 10% FBS (HyClone, Logan, UT, USA) and 5 µM Y-27632.

### 2.4. DNA Constructs

The following AAV Rep-Cap plasmids were used in the study: pAAV-RC1 (Cell Biolabs, San Diego, CA, USA), pAAV-RC2 (Agilent, Santa Clara, CA, USA), pAAV-RC6 (Cell Biolabs), pAAV2/9n (Addgene #112865), and pAAV-DJ (Cell Biolabs, San Diego, CA, USA). Plasmids pRC-Myo2A and -Myo4E were generated by site-directed mutagenesis of the pAAV2/9n construct.

The expression vector pAAV-CMV-eGFP was previously described [37]. The CMV promoter was replaced by sequences of the muscle or cardiac promoters. The promoter sequences and details of the cloning procedures are available in the Supplementary Information File, Table S1.

To generate the pAAV-cTnT-eGFP-amiR-A12.1 vector, the sequence of the 22-mer guide strand (A12.1), directed to the pathogenic *FLNC* variant c.7416_7418delGAA, was provided by Marlin Biotech LLC (Sochi, Russia). The artificial miRNA (amiR-A12.1) was created in the backbone of the endogenous pri-hsa-miR-30a. The amiR-A12.1 insert was cloned into the 3′ UTR of the pAAV-CMV-miR cloning vector as previously described [38]. The CMV promoter was replaced by the cTnT promoter sequence, resulting in the pAAV-cTnT-eGFP-amiR-A12.1 vector.

### 2.5. Viral Vector Preparation

Adeno-associated virus vectors were produced and quantified as genomic copies (GC) per mL by the Viral Vector Core of Marlin Biotech LLC (Sochi, Russia) [37,39,40].

### 2.6. AAV Cell Transduction

The cell cultures grown in a 24-well plate were transduced with AAV vectors with a multiplicity of infection (MOI) of 10^6^ GC per cell (roughly 10^11^ GC per mL per well), which is higher compared to MOI reported in previous studies [41,42,43]. Cells treated with DPBS (vehicle) served as a negative control. The growth medium was replenished with fresh complete medium every two days post-transduction. The cells were incubated for 3–17 days depending on the cell line, as detailed in the text and figure legends.

### 2.7. Immunofluorescence Imaging of iPSC-CMs

The iPSC-CMs were washed with HBSS buffer (Paneco, Moscow, Russia), fixed in 4% paraformaldehyde (PanReac, Barcelona, Spain) for 15 min, washed with HBSS, and followed by permeabilization with 0.2% Triton X-100 at room temperature for 10 min. Cells were washed three times and blocked with 0.1% Tween-20 (PanReac, Barcelona, Spain) in PBS + 5% goat serum (Gibco, Grand Island, NY, USA) and 5% FBS (HyClone, Logan, UT, USA) (PBS-T) for 30 min. Cells were incubated with mouse monoclonal anti-cTnT antibodies (1:1000, #4T19cc, HyTest, Turku, Finland) at +4 °C overnight. After washing with PBS-T, cells were stained with goat anti-mouse secondary antibody, Alexa Fluor 555 (1:800, #A28180, Invitrogen, Carlsbad, CA, USA) or goat anti-mouse secondary antibody, Alexa Fluor 488 (1:800, #A-11001, Invitrogen, Carlsbad, CA, USA) and F-actin stainer, Alexa Fluor 488 Phalloidin (1:400, #A12379, Invitrogen, Carlsbad, CA, USA) in PBS-T at room temperature for 30 min. Cells were washed two times with PBS-T for 5 min and incubated in 100 ng/mL DAPI (Sigma-Aldrich, St. Louis, MO, USA) for 10 min in the dark, washed once with PBS, and stored in PBS with 0.02% sodium azide. Images were acquired with a fluorescent microscope, Olympus IX53F (Olympus, Tokyo, Japan), operated by cellSens (Olympus, Tokyo, Japan). The images were processed by Fiji (ImageJ 1.54f).

### 2.8. Flow Cytometry

Cells were detached with trypsin-EDTA solution (0.05%), washed in DPBS, and centrifuged at 300× *g* for 5 min at 4 °C. The pellet was resuspended in DPBS supplemented with 10% FBS. To quantify the fraction of eGFP-positive cells and the integral fluorescence, the flow cytometry analysis was performed with gating at 525 nm on a Beckman Coulter Cytoflex S flow cytometer (Beckman Coulter Inc., Indianapolis, IN, USA). Untreated cells were used to set up autofluorescence thresholds. The cell count vs. eGFP intensity plots were prepared using Beckman Coulter CytExpert 2.4 software (Beckman Coulter Inc., Indianapolis, IN, USA).

### 2.9. AAV Administration in Mice

All AAV injections performed on 8–10-week-old mice. Prior to AAV injections, mice were anesthetized with a tiletamine hydrochloride/zolazepam hydrochloride mix (50 mg/kg) and xylazine (7 mg/mL) via intraperitoneal injections unless indicated otherwise.

The B10 mice (*n* = 20) were used in the study on AAV administration routes. For indirect intracoronary (IC) injections, animals (*n* = 5) were placed supine and ventilated with isoflurane (1.5%) vaporized in 100% O_2_ using a volume-controlled ventilator (Harvard Apparatus, Holliston, MA, USA). The chest cavity was opened along the subclavian line between the fourth and fifth ribs, and then a vascular clamp was used to occlude both the proximal aorta and the pulmonary artery. A 31-gauge needle was advanced into the left ventricular cavity to deliver 5 × 10^11^ GC per heart per animal in a volume of 50 μL. The AAV vector was delivered as a bolus immediately after cross-clamping, and the clamp was maintained for 45 s after vector injection. After releasing the vascular clamp, the chest and skin were closed with silk sutures. For direct intramyocardial (IM) injection, a thoracotomy was performed along the left subclavian line between the fourth and fifth ribs, the pericardium was opened, and AAV vectors at 5 × 10^11^ GC per heart per animal in 50 μL were administered into the left ventricle (*n* = 5). Finally, the intercostal incision was closed in layers with a 6.0 absorbable suture, the endotracheal tube was removed, and spontaneous breathing was restored. For intravenous (IV) injections, mice (*n* = 5) received injections of the vector at 4 × 10^12^ GC per animal (~2 × 10^14^ GC kg^−1^) diluted in 300 µL DPBS into the retroorbital venous sinus, 150 µL per each sinus. Mice treated intravenously with DPBS were used as a control group.

A total of *n* = 12 mdx mice participated in the serotype study and were subjected to IV injections of AAV at a dose of 8 × 10^13^ GC kg^−1^ (*n* = 3 per serotype or DBPS control). The promoter testing was performed on B6 mice (*n* = 20 total animals); AAV constructs were administered via IV injections at a dose of 4 × 10^13^ GC kg^−1^ (*n* = 5 animals per construct or DPBS control). The MyoAAV 2A-cTnT-eGFP-amiR-A12.1 vector was administered via IV injections into B6 mice at a dose of 2 × 10^14^ GC kg^−1^ (*n* = 7 per vector or DBPS control).

### 2.10. Tissue Collection

Four weeks post-injection, mice were euthanized with an overdose of anesthesia. Whole organs were briefly visualized on a Vilber Lourmat TFX-20 MC transilluminator (Vilber Lourmat, Collégien, France). For vector biodistribution and gene expression studies, the specified tissue samples were collected and snap-frozen in liquid nitrogen. Samples were stored at −70 °C before analysis. For immunofluorescent staining, tissues were embedded in Tissue-Tek OCT Compound mounting medium (Sakura Finetek, Tokyo, Japan) and frozen in isopentane (PanReac, Barcelona, Spain) pre-cooled with liquid nitrogen. Samples were stored at −70 °C before analysis. For imaging of whole heart and liver sections, anesthetized mice were subjected to cardiac perfusion with 10% buffered formalin, followed by organ isolation and fixation in formalin for 24 h. Free-floating sections were stored in 1x PBS supplemented with 0.1–0.2% (w/v) sodium azide at 4 °C.

### 2.11. Fluorescent Microscopy

A series of transverse 50-μm-thick sections of the formalin-fixed whole mouse heart and liver were prepared on the vibratome Microm HM-650V (Thermo Fisher, Waltham, MA, USA). Images were acquired using a Stellaris 5 confocal microscope (Leica Microsystems, Wetzlar, Germany) equipped with a Plan-Apochromat 10x objective. Cryosections (10 µm) were obtained using a CM 1510-1 cryostat (Leica) as previously described [44]. Sections were fixed on slides in 4% PFA with 2% sucrose. Images were acquired on a Stellaris 5 confocal microscope. The acquisition settings were adjusted using samples from DPBS-treated groups and were identical for all experimental groups. Figure panels were assembled in GIMP 2.10.34.

The images of the cells were obtained with the Carl Zeiss Axiovert 200M system and the LOMO TC-500 digital camera operated by LOMO MC-Foto 2.6 software and the Carl Zeiss N-Achroplan 10x/0.25 objective (Carl Zeiss, Oberkochen, Germany). The images were taken under equal conditions (sensitivity 65, exposure 50).

The mean eGFP signal intensity was quantified by the normalization of the integral eGFP signal intensity of the tissue to the area of the tissue and subsequent subtraction of background fluorescence. CellProfiler 4.2.8 software was used for the analysis.

### 2.12. AAV Biodistribution

Quantification of AAV vector DNA in tissues was performed by ITR-specific TaqMan qPCR as previously described [38] and presented as genomic copies (GC) per 1 μg of tissue DNA.

### 2.13. eGFP mRNA Quantification

Total RNA was isolated from powdered tissues using TRIzol Reagent (15596026, Thermo Fisher, Waltham, MA, USA) supplemented with 1-bromo-3-chloropropane (B9673, Sigma) following the manufacturer’s protocol [45]. Purified RNA (2 μg) was treated with RNase-free DNase I (EN0521, Thermo Fisher, Waltham, MA, USA) and reverse transcribed using an MMLV-RT Kit (SK021, Evrogen, Moscow, Russia) with a mixture of 1 μM random (dN)10 (SB002, Evrogen) and 1 μM oligo (dT)_15_ (SB001, Evrogen) primers.

For eGFP mRNA quantification in murine tissues, TaqMan real-time PCR reactions were set up using master mix (M-428, Synthol, Moscow, Russia), supplemented with 3 mM MgCl_2_, and *eGFP*-specific 400 nM primers (EGFP-120F: GACTGGGTGCTCAGGTAGTG, EGFP-120R: CAAGATCCGCCACAACATCG) and 200 nM probe (EGFP-FAM: FAM-TGTTCTGCTGGTAGTGGTCGGC-BHQ1). The 10-fold serial dilutions of the reference plasmid pAAV-CMV-eGFP were used to build the standard curve and calculate copies of eGFP transcripts per 100 ng of total RNA. The expression of housekeeping genes was analyzed for data normalization. The TaqMan multiplex reaction was set up using master mix (M-428, Syntol, Moscow, Russia) supplemented with 5 mM MgCl_2_ and 400 nM primers and 200 nM probes specific to *Rpl13a* (forward: CAACGGACTCCTGGTGTGAA, reverse: GTGCGCTGTCAGCTCTCTAA, probe: FAM-AAAGACTGTTTGCCTCATGCCTGC-BHQ1), *Ap3d1* (forward: GCCCAGCGTGTGGACATTAT, reverse: GCCAGGGGTTTATCCAGGTC, probe: ROX-CACTGAGGAGATGCCAGAGAATGCTT-BHQ2), and *Csnk2a2* (forward: GAGCTTGGGCTGCATGTTA, reverse: CCCAGAACCTTGGCAATTCG, probe: VIC-TTCCACGGGCAGGACAACTATGAC-BHQ1). All amplifications were performed on the CFX96 Real-Time PCR Detection System (Bio-Rad, Hercules, CA, USA).

### 2.14. Artificial miRNA Quantification

Detection of the mature guide strand of the artificial miRNA amiR-A12.1 was performed by stem-loop RT-qPCR, similar to the previously described procedure [38]. Briefly, RNA was extracted by standard protocol, and the reverse transcription was performed with the MMLV RT kit (SK021, Evrogen, Moscow, Russia) in the presence of 30 nM amiR-A12.1-specific stem-loop primer (5′-GAAAGAAGGCGAGGAGCAGATCGAGGAAGAAGACGGAAGAATGTGCGTCTCGCCTTCTTTCTCCCCCAC-3′). The resulting cDNA was amplified using 200 nM amiR-A12.1-specific forward primer (5′-GAAGGCGAGGAGCAGATCG-3′) and 200 nM universal reverse primer (5′-GCGGTAGTGGACGCCATC-3′) in 1x SYBR Green PCR master mix (PK147L, Evrogen). The reactions were carried out in the CFX96 Real-Time PCR Detection System (Bio-Rad Laboratories) with the following program: 95 °C-3 min; 39 cycles: 95 °C-10 s, 64 °C-15 s, 72 °C-15 s; 72 °C-5 min. A synthetic 22-mer RNA guide strand (5′-UAGUGGACGCCAUCGUGGGGGA-3′) served as a standard; 10-fold serial dilutions were used to calculate the standard curve. The copy number of amiR-A12.1 in samples was quantified and normalized to U6 expression.

### 2.15. eGFP Protein Microplate Assay

Murine tissue samples were lysed in RIPA buffer. For each sample, 700 ng of total protein was transferred to a 96-well plate. The measurement of eGFP fluorescence was performed on the CLARIOstar Plus Microplate Reader (BMG Labtech, Ortenberg, Germany) with a 525-nm filter.

### 2.16. Statistical Analysis

Statistical analysis was performed using OriginPro 2022 software with the Mann–Whitney test. The number of biological replicates for each experiment is indicated in the figure legends.

## 3. Results

### 3.1. Comparison of AAV9 Administration Routes for Uniform Transduction of Mouse Heart

In the pilot experiment, we compared local and systemic AAV administration modalities for cardiac transduction in mice. Several studies suggested that local AAV injections, such as indirect intracoronary (IC) and direct intramyocardial (IM), are preferred methods for cardiac gene transfer [46,47,48]. Local heart injections require significantly lower doses but are invasive and technically challenging in small animal models. By contrast, systemic intravenous (IV) infusions are widely used for AAV treatment in mice, yet require higher dosing as the vector is immediately diluted by the blood and distributed to off-target tissues [24].

**Figure 1:**
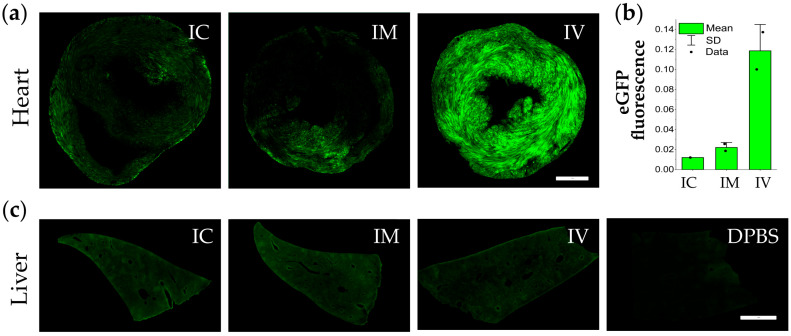
AAV-mediated transgene expression in the murine heart and liver under different administration conditions. The adult wild-type mice were treated with the AAV9-CMV-eGFP vector by indirect IC (5 × 10^11^ GC/animal), IM (5 × 10^11^ GC/animal), and IV (4 × 10^12^ GC/animal) injections (*n* = 5 animals per treatment). Four weeks post-injection, eGFP expression in the heart and liver sections was analyzed by fluorescent microscopy. (**a**) Representative images of heart sections following each injection condition are shown. Scale bar: 1 mm. (**b**) Semi-quantitative evaluation of relative eGFP signal intensity of the heart sections after IC (*n* = 1 sample), IM (*n* = 2), and IV (*n* = 2) injections. (**c**) Representative images of liver sections are shown. Scale bar: 1 mm.

We used common myotropic AAV serotype 9 (AAV9) and AAV-CMV-eGFP expression vector encoding the enhanced green fluorescent protein (eGFP) under the ubiquitous cytomegalovirus (CMV) promoter. Based on literature analysis, adult mice were injected with a high AAV dose for each administration route: 5 × 10^11^ GC per animal for IC and IM heart injections [46,47,49] and 4 × 10^12^ GC per animal (which corresponds to 2 × 10^14^ GC/kg) for systemic IV injections [13,21,25]. Four weeks post-injection, AAV9 transduction efficiency was assessed by the fluorescent confocal imaging of the heart sections; the liver served as a non-target organ (Figure 1). As evidenced by the eGFP signal, all types of injections provided successful heart transduction and transgene expression, albeit with different efficiency and distribution patterns (Figure 1a). Following IC injections, eGFP expression was primarily observed in epicardial myocytes of the outer heart surface, while penetration into the inner layers of myocardium was limited. For direct IM injections, the strongest eGFP signal was typically observed in the direct vicinity of the injection site; transgene expression in other areas of the heart was poor, in line with some previous reports [50]. The IV injection resulted in uniform eGFP expression across the whole heart section. Moreover, the intensity of the eGFP signal in heart sections was higher for systemic injections, surpassing heart-localized injections (Figure 1b). Surprisingly, a similar transduction level was seen in the liver regardless of delivery route (Figure 1c), confirming previous reports [46,48].

A serious limitation of our pilot experiment is that eGFP expression was examined in a small number of sections; serial heart sectioning and eGFP mRNA quantification were not performed. Nevertheless, our data demonstrates uniform cardiac transduction following IV injection and supports the trend for non-invasive systemic AAV administration for cardiac delivery in mice [6,7,25]. However, transduction of the non-target organs remains a major issue.

### 3.2. MyoAAV 2A Outperforms Native AAV9 in Heart Tropism Following Systemic Administration

**Figure 2:**
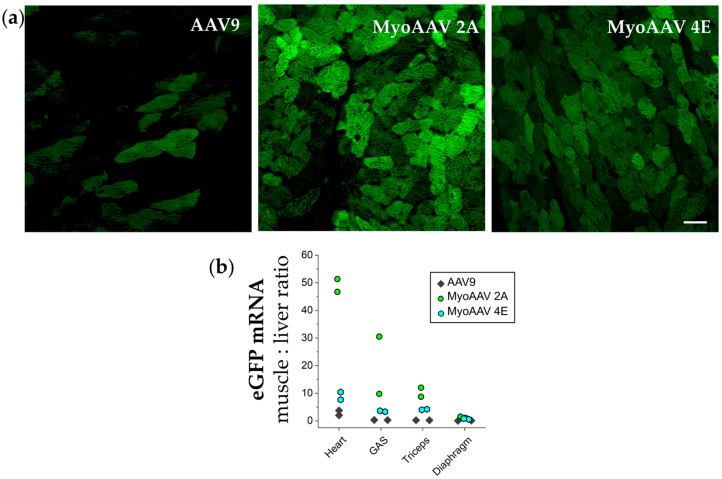
Direct comparison of MyoAAV 2A and 4E serotypes with parental AAV9 for heart-liver transduction properties. The 8-week-old *mdx* mice were systemically injected with AAV9-CMV-eGFP, MyoAAV 2A-CMV-eGFP, and MyoAAV 4E-CMV-eGFP vectors at 8 × 10^13^ GC/kg (*n* = 3 animals per treatment). Four weeks post-injection, eGFP fluorescence (*n* = 1) and mRNA levels (*n* = 2) were evaluated. Cryosections of mouse hearts were analyzed by fluorescent microscopy. (**a**) Representative images show transduction of murine myocardium by serotypes AAV9, MyoAAV 2A, and MyoAAV 4E. Scale bar: 30 μm. (**b**) The expression of eGFP mRNA in muscles and liver was quantified by RT-qPCR and presented as a muscle-to-liver ratio for each animal. Each data point represents one sample.

Next, we aimed to choose an AAV serotype with the improved cardiac-to-liver transduction properties. For testing, we preselected AAV9-based capsid variants MyoAAV 2A and MyoAAV 4E, which were developed by directed evolution for muscle-directed gene delivery [21]. To ensure the comparability of results, we took advantage of the *mdx* mouse model, which was utilized by Tabebordbar and coauthors to assess the potential therapeutic relevance of the MyoAAV 2A capsid. Considering the enhanced potency of engineered serotypes, the adult mice were IV injected with MyoAAV 2A-, MyoAAV 4E-, and native AAV9-CMV-eGFP vectors at the lowered dose of 8 × 10^13^ GC/kg to minimize the possibility of signal hyperintensity. Four weeks after administration, the efficiency of heart transduction coupled with tropism to non-target organs was evaluated. All vectors successfully transduced murine hearts, providing eGFP signal (Figure 2a) and comparable levels of eGFP mRNA (Figure S1) in cardiomyocytes. We assessed the relative tropism of AAV vectors to cardiac and other skeletal muscles (gastrocnemius (GAS), triceps, and diaphragm) by normalizing eGFP mRNA levels in each organ to corresponding values in the liver (Figure 2b). Both MyoAAV variants surpassed the myotropic properties of AAV9 in the heart and skeletal muscles, as was previously reported [21]. Importantly, MyoAAV 2A showed enhanced cardiac tropism, demonstrating the ~50-fold higher eGFP mRNA levels in the murine heart over the liver. The corresponding heart:liver ratio was ~9 for the MyoAAV 4E variant and ~3 for the native AAV9 serotype (Figure 2b). Our gene expression data are consistent with the vector genome biodistribution performed by Tabebordbar and coauthors (2021), which revealed significantly lower tropism of MyoAAV 2A to the liver compared to AAV9.

Therefore, we confirmed the improved heart-to-liver tropism of MyoAAV 2A and selected this capsid variant for further studies.

### 3.3. Screening of Muscle- and Cardiac-Specific Promoters In Vitro

**Figure 3:**
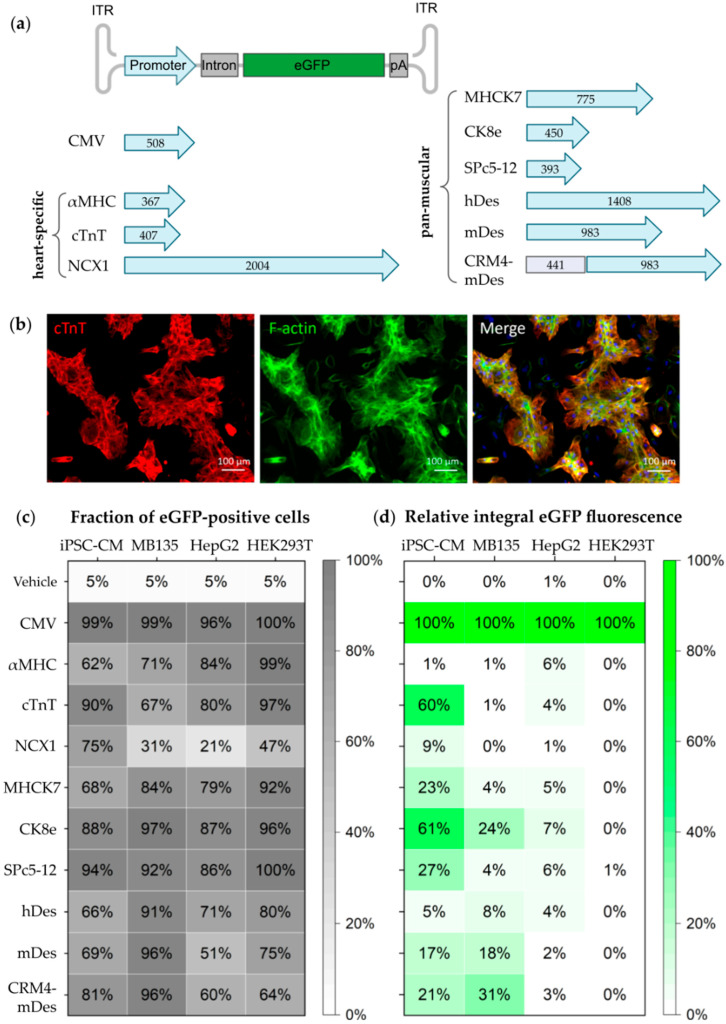
In vitro evaluation of commonly used cardiac and pan-muscular promoters to drive efficient and selective expression in cardiomyocytes. (**a**) Schematically shown is the AAV expression vector with the reporter eGFP under the control of one of the selected promoters. CMV, human cytomegalovirus immediate-early enhancer and promoter; αMHC, promoter of α-myosin heavy chain; cTnT, promoter of chicken cardiac troponin T gene; NCX1, promoter of feline cardiac Na+/Ca^2+^ transporter; MHCK7, hybrid promoter, conjugate of creatinine kinase regulatory region and α-myosin heavy chain enhancer; SPc5-12, synthetic promoter, combination of SRE, TEF-1, MEF-1, and MEF-2 regulatory sites; CK8e, hybrid promoter based on muscle creatinine kinase gene; mDes, murine desmin promoter; hDes, human desmin promoter; CRM4-mDes, murine desmin promoter with CRM4 enhancer. The size in base pairs is indicated for each promoter. (**b**) Patient-specific hiPSC-CMs (line IPSFIL14S) served as an in vitro model for screening promoter activity. hiPSC-CMs were co-immunostained for the cardiac marker cTnT and cytoskeleton protein F-actin; nuclei were counterstained with DAPI (blue). Images demonstrate colocalization of cTnT and actin filaments and confirm successful differentiation. The iPSC-CMs and non-cardiac immortalized cell lines were transduced with AAV-DJ vectors encoding the eGFP gene under the control of various promoters (MOI of 10^6^ GC/cell). Seven days post-transduction, the percent of eGFP-positive cells (**c**) and the integral eGFP fluorescence (**d**) in each cell line were quantified by flow cytometry (see Figure S6). The level of eGFP fluorescence achieved with the CMV promoter was set as 100%.

Despite the enhanced cardiac tropism of MyoAAV 2A, CMV promoter-driven expression of the transgene in the liver and skeletal muscles remained substantial. To restrict transgene synthesis to cardiac tissues, we next optimized promoter elements within the AAV expression cassette (Figure 3a). The first group of promoters selected for the study included murine αMHC [51,52], chicken cTnT [53,54,55], and feline NCX1 [56,57]. These promoters have been reported to exhibit varying degrees of cardiac-specific activity, as demonstrated in multiple studies, including a recent high-throughput analysis [58]. The second group included pan-muscle-specific promoters murine MHCK7 [59], synthetic chicken SPc5-12 [60]), murine CK8e [61], murine desmin (mDes) [62], and human desmin (hDes) [62]. These promoters are well characterized and already used in AAV-based gene therapies undergoing clinical trials for muscle disorders such as Duchenne muscular dystrophy, myotubular myopathy, Pompe disease, and others [27]. Additionally, we included in the panel an improved version of the mDes promoter with the CRM4 enhancer (CRM4-mDes) [62], which was shown to increase cardiac transcription [63]. To the best of our knowledge, a side-by-side comparison of such a cardiac and pan-muscular promoter panel has not yet been performed. To create a set of “sibling” expression vectors, we replaced the CMV-promoter in the parental plasmid pAAV-CMV-eGFP with one of the selected promoter sequences (Figure 3a and Table S1).

As a cellular model for screening candidate promoters, we used human induced pluripotent stem cell-derived cardiomyocytes (iPSC-CMs). The iPSC line IPSFIL14S (Figures S2 and S3) was generated from the dermal fibroblasts of a patient with a cardiac genetic disorder (see Supplementary Materials and Methods). The obtained iPSC-CMs represent beating cardiomyocytes with a robust expression of the cardiac troponin T (cTnT) protein marker, co-localizing with actin filaments (F-actin) (Figure 3b and Figure S4). Fluorescence microscopy also indicates high homogeneity of the culture, but the % of cTnT+ cells was not assessed by flow cytometry.

Given the difference in AAV transduction efficiency in vivo and in vitro [41], we next determined the suitable AAV serotype to deliver the expression cassette into the cardiomyocyte-enriched cell culture. We transduced control iPSC-CMs derived from the iPS12 line with natural serotypes AAV1, AAV2, AAV6, AAV9, and synthetic AAV-DJ, widely used for in vitro screening [42,43,64]. The CMV-driven eGFP expression was evaluated by fluorescent microscopy, Western blot, and flow cytometry (Figure S5). Both AAV6 and AAV-DJ capsids appeared to be equally efficient, transducing up to 97% of cardiomyocyte-enriched cells at an MOI of 10^6^ GC/cell. While AAV6 has long been studied for human iPSC-CM transduction [42,43,58], high effective doses of AAV6 appeared toxic at least in some cell types [40,65]. For our in vitro experiment we selected AAV-DJ, a hybrid of AAV2, AAV8, and AAV9 capsids, with remarkably high infectivity across a wide range of cell types [64,66].

The selected promoters (αMHC, cTnT, NCX1, MHCK7, CK8e, Spc5-12, mDes, hDes, and CRM4-mDes) were vectorized in the AAV-DJ serotype for testing their efficiency and selectivity in vitro. The vector with a CMV promoter was used as a reference to monitor transduction efficiency and ubiquitous eGFP expression. The patient-specific iPSC-CMs were transduced with AAV-DJ vectors (MOI of 10^6^ GC/cell), and promoter-driven eGFP transcription in cardiac cells was analyzed 7 days post-transduction (Figure S6). To quantify the eGFP signal, we performed flow cytometry of AAV-transduced iPSC-CMs (Figure S6) and analyzed both the percent of eGFP-positive cells and mean fluorescence. The AAV-DJ transduction efficiency of patient-specific cardiomyocytes was nearly 100%, based on the percentage of eGFP-positive cells following treatment with a CMV-driven vector (Figure 3c and Figure S6).

Cardiac and pan-muscular promoters exhibited highly variable activity in the cardiomyocyte-enriched cell model compared to the ubiquitous CMV promoter. Promoters cTnT and SPc5-12 were active in >90% of transduced iPSC-CMs, followed by CK8e (88%), CRM-mDes (81%), and NCX1 (75%) promoters (Figure 3c). The proportion of eGFP-positive cells was below 70% in iPSC-CMs transduced with vectors containing αMHC, MHCK7, hDes, and mDes, indicating that promoter activity was not supported in all AAV-transduced cells. As for the average eGFP fluorescence, the highest level was detected for cTnT and CK8e promoters, ~60% of the CMV-driven expression (Figure 3d). The MHCK7, Spc5-12, and CRM4-mDes induced lower levels of eGFP signal in the range 20–30% of CMV-driven expression. Unlike previous studies [58], cardiac promoters αMHC and NCX1 showed low activity in cardiomyocyte-enriched culture, below 10% of the CMV-driven eGFP signal. An apparent limitation of our experimental design is that co-localization of eGFP with cardiomyocyte markers and absence in non-cardiomyocytes was not assessed.

The selectivity of promoter activity was further tested in non-cardiac immortalized cell lines: MB135, human myoblasts; HepG2, human hepatocarcinoma cells; and HEK293T, human embryonic kidney epithelial cells (Figure S7). The transduction efficiency of immortalized cell lines with AAV-DJ was 100%, as demonstrated by a control vector with the ubiquitous CMV promoter (Figure 3c). Expectedly, the activity of tissue-specific promoters was lower in the immortalized cell lines compared to the control CMV promoter (Figure 3d). In MB135 myoblasts, the highest eGFP expression was induced by pan-muscular promoters CK8e, mDes, and CRM4-mDes (24%, 18%, and 31% of CMV-driven expression, respectively). Other promoters, including cardiac cTnT, induced less than 10% of eGFP signal intensity. In non-muscular cell lines HepG2 and HEK293T, all tested promoters induced less than 10% of CMV-driven eGFP expression (Figure 3d).

For in vivo testing, we selected cardiac cTnT and pan-muscular CK8e promoters, which ensured a high level of transgene expression in iPSC-derived cardiomyocytes in the screening experiment. Despite the modest in vitro data, we also selected the CRM4-mDes promoter, which previously demonstrated increased expression in the heart and other muscle groups in mice [62,63].

### 3.4. Evaluation of cTnT, CK8e, and CRM4-mDes Promoters for Cardiac-Specific Expression in Mice

**Figure 4:**
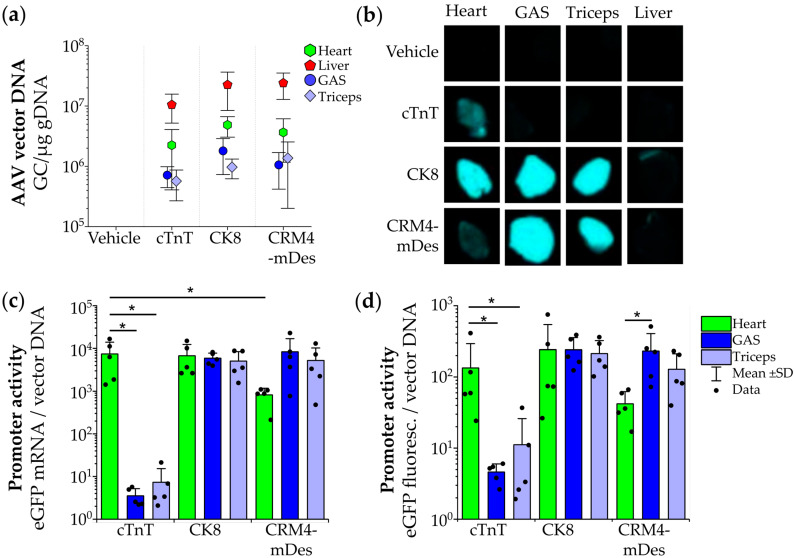
The activity of cardiac and muscular promoters in the heart and skeletal muscles following systemic MyoAAV 2A-mediated delivery. Adult mice were intravenously injected with 4 × 10^13^ GC/kg of MyoAAV 2A vectors carrying eGFP reporters under cTnT, CK8e, or CRM4-mDes promoters and analyzed four weeks after injection. (**a**) MyoAAV 2A vector biodistribution in murine tissues. Data presented as mean ± SD, *n* = 5 animals per group. For all vectors, the difference in vector distribution between similar tissues was statistically insignificant, as established by the Mann–Whitney test. (**b**) eGFP fluorescence in different tissues of AAV-treated mice illuminated by blue light. (**c**) The relative promoter activity calculated as eGFP mRNA expression quantified by RT-qPCR (see Materials and Methods and Figure S8a), normalized to the AAV vector DNA; the promoter activity in the liver was set as 1. (**d**) The relative promoter activity calculated as eGFP fluorescence measured in the tissue lysates by the microplate assay, normalized to AAV vector DNA. Data presented as organ-to-liver ratio. * *p* < 0.05 (Mann–Whitney test).

Next, we tested cTnT, CK8e, and CRM4-mDes promoters in murine hearts and off-target tissues in vivo. The eGFP reporter constructs were packaged in MyoAAV 2A serotype, which demonstrated improved heart-to-liver tropism in mice (Figure 2). To avoid eGFP signal saturation, we further decreased the AAV dose to 4 × 10^13^ GC/kg, compared to 2 × 10^14^ GC/kg in the administration route experiment and 8 × 10^13^ GC/kg in the serotype selection experiment. Eight-week-old C57BL/6J mice were intravenously injected with MyoAAV 2A vectors, followed by quantification of vector DNA distribution and transgene expression in tissues four weeks post-injection (Figure 4). All three MyoAAV 2A vectors had similar biodistribution in murine tissues (Figure 4a). The highest content of AAV vector genomes was detected in the liver (1.1–2.4 × 10^7^ GC per 1 μg genomic DNA), followed by the heart (2.2–4.9 × 10^6^ GC) and skeletal muscles (0.72–1.8 × 10^6^ GC). As evidenced by eGFP fluorescence, promoters cTnT, CK8e, and CRM4-mDes yielded detectable levels of the reporter transcription in murine hearts, but not in the liver (Figure 4b).

To evaluate the efficiency and selectivity of promoters, we quantified eGFP mRNA by RT-qPCR in the heart, skeletal muscles (gastrocnemius and triceps), and liver (Figure S8a). The highest level of eGFP transcripts was detected in the heart samples and ranged from 2.7 ± 1.2 × 10^5^ copies per 100 ng of total RNA for the CRM4-mDes promoter to 1.3 ± 0.8 × 10^6^ and 7.1 ± 3.1 × 10^6^ copies for the cTnT and CK8e promoters, respectively. Unlike pan-muscular CK8e and CRM4-mDes promoters, the cTnT promoter also provided negligible levels of reporter mRNA in the gastrocnemius and triceps (less than 600 copies per 100 ng mRNA, respectively). For all samples, the eGFP expression in the liver was 2–3 orders of magnitude lower than in the heart. To obtain values of relative promoter activity, we normalized eGFP mRNA expression in each tissue to the corresponding AAV vector DNA; the promoter activity in the liver was set as 1 (Figure 4c). The activity of both cTnT and CK8e promoters in the heart by 6000-fold exceeded the promoter activity in the liver. As previously shown [58,67], only cTnT promoters demonstrated cardiac-specific properties with activity in the skeletal muscles ~1000-fold lower compared to the heart. The pan-muscular promoter CK8e demonstrated comparable activity in the heart and the skeletal muscles, while the CRM4-mDes promoter was 6–10 times more active in the skeletal muscles than in the murine myocardium.

To assess promoter activity at the protein level, we quantified eGFP fluorescence in the tissue lysates by microplate assay (Figure S8b) and normalized to AAV vector DNA (Figure 4d). In line with mRNA data, only the cTnT promoter demonstrated cardiospecific activity 10 times higher than in skeletal muscles. The relative activity of the CK8e promoter in the heart and skeletal muscles did not differ significantly, while the CRM4-mDes promoter showed preference for skeletal muscles over the heart.

To summarize, following systemic delivery with the MyoAAV 2A serotype, the promoters CK8e, cTnT, and CRM4-mDes enabled efficient reporter transgene expression in the murine heart with minimal expression in the liver. However, only the cTnT promoter demonstrated cardiac-specific activity, with a strong preference for the myocardium over skeletal muscles. These data prove the superiority of cTnT as an optimal heart-specific promoter.

### 3.5. MyoAAV 2A Serotype and cTnT Promoter Enable Cardiac-Specific Expression of miRNA-Based Transgene in Murine Heart Following Systemic AAV Injection

**Figure 5:**
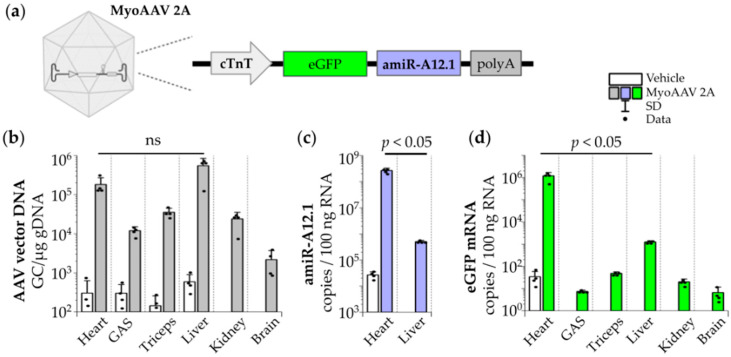
The artificial miRNA is expressed and processed in murine hearts following systemic injection of the MyoAAV 2A-cTNT-eGFP-amiR-A12.1 vector. (**a**) Schematically shown is the MyoAAV 2A vector carrying the expression cassette cTnT-eGFP-amiR-A12.1, which enables cTnT-driven synthesis of the protein-coding transgene (eGFP) and miR-30-based artificial miRNA (amiR-A12.1). The MyoAAV 2A-cTNT-eGFP-amiR-A12.1 vector was IV injected into adult, wild-type mice at 2 × 10^14^ GC/kg; *n* = 4 for the vector or vehicle (DPBS) group. (**b**) MyoAAV 2A vector biodistribution in murine tissues one month post-injection. (**c**) Expression of the 22-mer mature guide strand of amiR-A12.1 quantified by stem-loop RT-qPCR in the heart and liver. (**d**) Expression of eGFP mRNA quantified by RT-qPCR. Data were presented as individual data points with mean ± SD. Statistical significance was determined using the Mann–Whitney test.

Finally, we addressed the potential of systemic MyoAAV 2A-mediated delivery and cTnT-driven expression to produce therapeutically relevant transgenes in the hearts of the wild-type mice (Figure 5). As a transgene, we utilized a non-coding, artificial microRNA amiR-A12.1, designed to silence via the RNAi mechanism the human pathogenic *FLNC* variant (Figure S9), associated with restrictive cardiomyopathy [34]. The amiR-A12.1 insert was placed into the 3′UTR in the pAAV-cTnT-eGFP, creating the pAAV-cTnT-eGFP-amiR-A12.1 vector for packaging in the MyoAAV 2A serotype (Figure 5a). The eGFP reporter was retained in the expression cassette to further monitor gene transfer efficiency by reporter expression.

The wild-type mice were systemically treated with the MyoAAV 2A vector at a dose of 2 × 10^14^ GC/kg, followed by biodistribution and transgene expression analyses 4 weeks post-injection. Given the high AAV dose, we also added a basic safety study to the experiment design (Figure S10). Standard vital monitoring and body weight dynamics (Figure S10a) did not reveal severe adverse events. At the 4-week time point, the blood biochemistry markers ALT, AST, ALP, and creatinine were within the physiological norm and did not differ between control and AAV-treated groups (Figure S10b). The hearts of mice that received MyoAAV 2A-cTnT-miA12.1 showed no significant fibrosis features (Figure S10c). Otherwise, AAV treatment was well-tolerated in all animals.

The relative biodistribution of MyoAAV 2A-cTnT-eGFP-amiR-A12.1 in the murine tissues was similar to previously obtained data with the parental vector without amiR (compare Figure 4a and Figure 5b). However, absolute values for the AAV genomes detected per 1 μg of host DNA were lower, possibly due to the influence of the RNAi-based transgene on the stability and functional persistence of the AAV vector genome [68]. Specifically, ~1.8 × 10^5^ and ~5.5 × 10^5^ GC/μg DNA were observed in the heart and the liver, respectively. In the skeletal muscles and kidney, AAV genome levels ranged within ~1.2–2.6 × 10^4^ GC/μg DNA, and approximately 10 times lower levels of AAV genomes reached the brain (~2.1 × 10^3^ GC/μg DNA).

The expression of both transgenes, protein-coding eGFP and non-coding amiR, was analyzed at the RNA level by RT-qPCR (Figure 5c,d). The highest level of cTnT promoter-driven expression of eGFP was expectedly detected in murine hearts (~1.2 × 10^6^ transcript copies per 100 ng total mRNA), followed by the liver (~1.3 × 10^3^ copies), skeletal muscles, kidneys, and the brain (less than 100 copies, Figure 5d). Importantly, the cTnT promoter enabled expression and proper processing of the amiR-A12.1 as evidenced by the detected 22-mer mature guide strand (Figure 5c). The level of the processed amiR-A12.1 reached ~2.7 × 10^8^ copies per 100 ng of RNA in the heart. In the liver, amiR levels were significantly lower, yet still substantial (~5 × 10^6^ copies), indicating that complete cardiac specificity was not achieved.

To conclude, the combined properties of the myotropic serotype MyoAAV 2A and the cardiac cTnT promoter allowed efficient and selective expression of the amiR transgene in the hearts of the wild-type mice following systemic administration. However, the challenge of complete liver detargeting has not yet been fully resolved.

## 4. Discussion

The first AAV-based gene therapy for striated muscle disorder (Elevidys, developed by Sarepta Therapeutics) was approved by the FDA in June 2023 for treating Duchenne muscular dystrophy. Since then, several novel AAV gene therapies for cardiac conditions have advanced to the clinical trials, including TN-201 (by Tenaya Therapeutics) for hypertrophic cardiomyopathy, LX2020 (by Lexeo Therapeutics) for arrhythmogenic right ventricular cardiomyopathy, RP-A501 (by Rocket Pharma) for Danon disease, SRD-001 (by Sardocor) for heart failure with preserved ejection fraction, ASP2016 (by Astellas) for cardiomyopathy associated with Friedreich ataxia, and others [24]. Remarkably, the first RNAi-based AAV gene therapy for heart failure (YAP101 by Medley Therapeutics) successfully passed its first safety milestone in late 2025 (NCT06831825). Despite this, the AAV methodology for efficient and selective cardiac targeting—sparing skeletal muscles and other off-target organs—remains a subject of debate.

Here, we revised disparate preclinical data on AAV administration routes, cardiotropic serotypes, and tissue-specific promoters for targeted transgene delivery to the mouse heart. Our pilot study on AAV9 administration routes demonstrated the advantage of intravenous injections over heart-directed injection (intramyocardial and intracoronary) for efficient and uniform cardiac transduction (Figure 1). Although a small number of animals (*n* = 1–2) were used to obtain representative images, and regional quantification across major cardiac areas was not performed, our study supports the use of non-invasive systemic injections for cardiac delivery as in recent reports [6,7].

The drawback of systemic AAV administration is biodistribution to off-target tissues and high vector doses required to reach efficient concentrations in the heart. We tested the potential of the novel AAV9-based capsids MyoAAV 2A and 4E [21] to reduce vector dose and detarget the liver. As assessed by reporter gene expression (Figure 2 and Figure S1), the engineered serotype MyoAAV 2A systemically injected at 8 × 10^13^ GC/kg outperformed the natural AAV9 and showed enhanced cardiac transduction with reduced hepatic uptake. For the purpose of the current study, we selected MyoAAV 2A for cardiac transgene delivery. However, this capsid remains far from meeting the need for truly cardiac-specific gene therapy. Indeed, the reporter expression in the liver remained substantial, averaging at ~10^5^ mRNA copies per 100 ng total RNA (Figure S1). The persistence of extensive liver transduction is a key cause of immune response and hepatotoxicity following high-dose AAV administration in clinical trials. It would therefore be important to compare the performance of MyoAAV 2A with other recently discovered cardiac-tropic and liver-detargeted capsids [69,70,71,72].

Tissue-specific promoters are another powerful tool to minimize off-target transgene expression and improve the safety profile of the vector. We screened a panel of ten tissue-specific promoters in vitro using the AAV-DJ platform. Similar to the AAV6 serotype commonly used for cardiomyocyte transduction [42,43,58], the DJ serotype exhibits broad tropism and high transduction efficiency across diverse cell types [64], including the four cell lines used in this study. The in vitro selection indicated chicken cTnT, chimeric CRM4-mDes, and murine CK8e as the leading candidates (Figure 3). In vivo, all promoters provided cardiac expression with minimal activity in the liver (Figure 4). Among them, cTnT showed the highest specificity, with >6000-fold enrichment in heart versus liver (0.02% residual hepatic activity) and 10-fold higher activity compared to skeletal muscles. In our system, the chicken cTnT promoter outperformed previously reported values by Prasad et al. (2011). This difference can be at least partially explained by distinct methods used to quantify promoter activity. While Ref. [67] measured luciferase luminescence, our study directly quantified transcripts with normalization to vector copy number. Implementation of methods with simultaneous DNA and RNA detection would further increase accuracy of the promoter activity data [73,74].

It is worth noting that promoters tested in the current study are derived from different species, including chicken cTnT, mouse αMHC, and feline NCX1. It is generally accepted that orthologous promoters share conserved *cis*-regulatory elements (e.g., transcription factor binding sites) and retain cross-species promoter activity. For instance, the chicken cTnT promoter is active in rat and chicken cardiomyocytes [53], as well as in zebrafish embryos [75]. Similarly, the NCX1 cardiac promoter contains a highly conserved (>90%) proximal promoter region among feline, human, and rodent *NCX1* genes. This explains the activity of the feline NCX1 promoter in transgenic mouse models [76] and primary rat cardiomyocytes [77]. Conversely, the promoter length and coordinates relative to the transcription start site may dramatically affect promoter activity in different experimental setups [27,78,79]. To maintain data consistency, authors should avoid generalizing the results with promoters and instead provide the comprehensive promoter description, including species origin, promoter size, genome coordinate, and the exact sequence (as in Table S1).

Finally, we combined the optimized components—systemic delivery, the MyoAAV2A capsid, and the cardiac-specific cTnT promoter—into a single AAV platform and evaluated it in vivo for cardiac delivery of a miR-30-based transgene amiR-A12.1 (Figure 5). Although vector genomes accumulated preferentially in the liver, transgene expression was strongly enriched in the heart, both for eGFP mRNA and for amiR-A12.1. For both transgenes, the relation of the heart-to-liver transgene expression was almost three orders of magnitude and showed even greater enrichment relative to other non-target organs (skeletal muscles, kidneys, and brain). This demonstrated that a tissue-specific promoter can override biodistribution at the functional level. Even though hepatotoxicity was not revealed in our pilot study (Figure S10b), further safety studies are needed to address both possible heart- and liver-toxic effects of cardiotropic capsids and physiologically active transgenes like RNAi effectors. In future studies, using a lower effective dose would reduce the likelihood of adverse events. Moreover, removing eGFP from the expression cassette would also improve the AAV safety profile and enhance amiR expression, but this issue falls beyond the scope of the present study.

## 5. Conclusions

We believe that current research highlights the important challenges that accompany the development of heart-specific gene therapy. We propose a composition of an AAV-based platform that demonstrates efficient and selective delivery of transgenes to the heart. Despite promising results, complete liver detargeting remains a subject of future research.

## Data Availability

The data generated during this study are available within the published article and its Supplementary Files or from the corresponding author on reasonable request.

## Supplementary Materials

The following supporting information can be downloaded at: https://www.mdpi.com/article/10.3390/biotech15030055/s1, Supplementary Materials and Methods, Figure S1: The eGFP expression in the heart and non-target organs following systemic administration of AAV9, MyoAAV 2A, and MyoAAV 4E vectors in adult mdx mice (8 × 1013 GC/kg). Four weeks post-injection, tissue samples were collected, followed by RNA extraction and eGFP mRNA quantification by RT-qPCR. See the detailed description in the Materials and Methods section of the manuscript. Each data point represents an organ of a single anima; Figure S2: Molecular and genetic characterization of the patient-specific human iPSC line IPSFIL14S.; Figure S3: Functional characterization of the patient-specific human iPSC line IPSFIL14S.; Figure S4: Characterization of human iPSC-derived cardiomyocytes; Figure S5: Selecting AAV serotype and multiplicity of infection for efficient transduction of iPSC-derived cardiomyocytes. The control human iPSC line (iPS12) was subjected to differentiation into cardiomyocytes (iPSC-CMs). Cells were transduced with AAV vectors carrying CMV-eGFP expression cassettes and packaged in natural serotypes AAV1, 2, 6, or 9 or chimeric serotype DJ; multiplicity of infection (MOI) roughly corresponded to 10^6^ and 10^7^ GC per cell. Seven days post-transduction, eGFP expression was analyzed by fluorescent microscopy, Western blotting, and flow cytometry; Figure S6: Flow cytometry analysis of promoter-driven eGFP fluorescence in cardiomyocytes in vitro. Patient-specific iPSC-CMs (clone IPSFIL14S) were transduced with AAV-DJ vectors encoding the eGFP gene under the control of various promoters at MOI 10^6^ of GC/cell. The eGFP fluorescence was analyzed 7 days post-transduction; Figure S7: Promoter activity in non-cardiac cell lines. Immortalized cell lines of human myoblasts (MB135), human hepatocarcinoma (HepG2), and human embryonic kidney epithelium (HEK293T) were transduced with AAV-DJ vectors encoding the eGFP gene under the control of cardiac and muscular promoters (MOI of 106 GC/cell). A vector with the strong ubiquitous CMV promoter was used as a control. The eGFP expression was detected by fluorescent microscopy 14 days (MB135), 4 days (HepG2), and 3 days (HEK293T) post-transduction. The representative images are shown; Figure S8: Quantification of eGFP mRNA and fluorescence driven by cardiac and pan-muscular promoters in mice. Eight-week-old C57BL/6J mice were intravenously injected with MyoAAV 2A vectors carrying the eGFP reporter under the control of the cTnT, CK8e, or CRM4-mDesmin promoter (4 × 10^13^ GC/kg). Four weeks post-injection, eGFP mRNA and fluorescence were quantified in the heart, skeletal muscles, and liver; Figure S9: Preliminary data on allele-specific silencing of mutant *FLNC* by artificial microRNA amiR-A12.1. Human patient-specific iPSC-CMs (derived from clone IPSFIL14S, see Supplementary Materials and Methods) were transduced with AAV-DJ-cTnT-amiR-A12.1 or scrambled control vector AAV-DJ-cTnT-SCR at an MOI of 10^6^. Seven days after treatment, relative levels of wild type (wt) and mutant (ΔGAA) *FLNC* transcripts were quantified by TaqMan RT-qPCR with allele-specific probes. The transcript levels in the scrambled control samples were set as 100%; n = 2 independent iPSC differentiation and transduction experiments; Figure S10: Basic safety study following systemic administration of MyoAAV 2A-cTNT-eGFP-amiR-A12.1 into wild-type mice; Table S1: Promoter sequences used in the study.

## Abbreviations

The following abbreviations are used in this manuscript:

AAV	adeno-associated virus vector
amiR	artificial microRNA
eGFP	enhanced green fluorescent protein
GC	genomic copies
IC	intracoronary
IM	intramyocardial
IV	intravenous
iPSCs	induced pluripotent stem cells
iPSC-CMs	induced pluripotent stem cell-derived cardiomyocytes
MOI	multiplicity of infection
RNAi	RNA interference
