# Supplementary material for: AAV Vector Toolkit for the Delivery and Expression of the Artificial microRNA in the Murine Heart
Source: BioTech (Basel). 2026 Jul 17;15(3):55. doi: 10.3390/biotech15030055 (PMC13398323; doi:10.3390/biotech15030055)
Supplement: Supplementary file 1 [file biotech-15-00055-s001.zip › biotech-4355838-supplementary.pdf]

Supplementary Materials: AAV Vector Toolkit for the Delivery and Expression of the Artificial miRNA in the Murine Heart

Ivan I. Galkin, Viktoria V. Skopenkova, Maria Y. Shubina, Anna V. Polikarpova, Svetlana G. Vassilieva, Irina M. Savchenko, Olga S. Lebedeva, Daria V. Goliusova, Margarita Yu. Sharikova, Vladimir V. Gureev, Tatiana N. Malorodova, Alexey V. Deikin, Tatiana V. Egorova and Maryana V. Bardina

1. Supplementary Materials and Methods

DNA constructs with cardiac and muscular promoters

A set of "sibling" expression vectors was created by replacing the CMV promoter in the parental plasmid pAAV-CMV-eGFP with one of the cardiac- or pan-muscle-specific promoter sequences. Promoter sequences and descriptions are detailed in Table S1. The chicken cTnT and feline NCX1 promoter sequences were synthesized by GenScript (China). The promoter sequences of murine αMHC, mDes, hDES, and human CRM4-enhancer were amplified from the genomic DNA of the corresponding species. Previously described constructs were used as a source of SPc5-12 and MHCK7 promoters [PMID: 35039573]. For all promoters, sequence amplicons were obtained by PCR with the forward and reverse primers containing BcuI and SacI (or BcuI and MluI for CRM4) restriction sites at the 5' end. Similarly, the CMV promoter sequence in the pAAV-CMV-amiR-A12.1 vector was replaced with the cTnT promoter by BcuI and SacI restriction cloning, generating the pAAV-cTnT-eGFP-amiR-A12.1 vector.

. Generation of patient-specific iPSC line iPSFIL14S

The iPSC line IPSFIL14S was reprogrammed from the dermal fibroblasts of the patient with restrictive cardiomyopathy caused by the heterozygous mutation c.7416_7418delGAA of the *FLNC* gene and characterized as previously described for iPSC line IPSFIL24S52 [PMID: 40806381]. Briefly, skin fibroblasts of the 3rd passage were reprogrammed via a non-integrative approach using the CytoTune-iPS Reprogramming Kit (ThermoFisher Scientific) according to the manufacturer's protocol. The emerging colonies were picked manually and transferred to the wells of a 24-well culture plate (1 clone per well) precoated with hESC-qualified Matrigel (Corning) in mTeSR1 medium (Stemcell Technologies) supplemented with 10 µM ROCK kinase inhibitor Y-27632 (Stemcell Technologies). The following daily medium exchanges were performed with mTeSR1 without Y-27632. In 4–6 days and further, upon reaching 70–80% confluence, iPSC clones were passaged with 0.05% trypsin-EDTA solution (Gibco). The pool of reprogrammed cells and resulting iPSC clones were banked and stored in liquid nitrogen. Several iPSC clones, including IPSFIL14S, were analyzed for karyotype stability, genetic identity, and pluripotency via differential G-staining of metaphase chromosomes, STR analysis, Sanger sequencing, RT-PCR, flow cytometry, and immunocytochemical staining methods as previously described [PMID: 40806381].

Western blot analysis

Cell lysate preparation and western blot procedures were performed as previously described [PMID: 35964317]. Antibodies used in the study: anti-GFP (1:4500, G1544-100UG, Sigma) and anti-GAPDH (1:10000, ABS16, Merck); and the HRP-labeled anti-rabbit IgG antibodies (1:3000, 170-6515, Bio-Rad). Membranes were incubated with Clarity ECL (Bio-Rad) and visualized with iBright (Thermo Scientific). A mild stripping procedure was used for reprobing membranes. The densitometric analysis of band intensity was performed by FIJI software.

Blood biochemistry

Alanine transaminase (ALT), aspartate aminotransferase (AST), alkaline phosphatase (ALP) and creatinine levels were evaluated in veterinary laboratory "Artvet", Moscow, Russia

Histopathology

The organs were weighed, fixed in 10% buffered formalin, and embedded in paraffin. Five-micron-thick transverse sections were stained with hematoxylin and eosin (H&E) as previously described [PMID: 28748459]. Images were acquired with a Nikon Eclipse Ti-E at 200× magnification.

2. Supplementary Figures

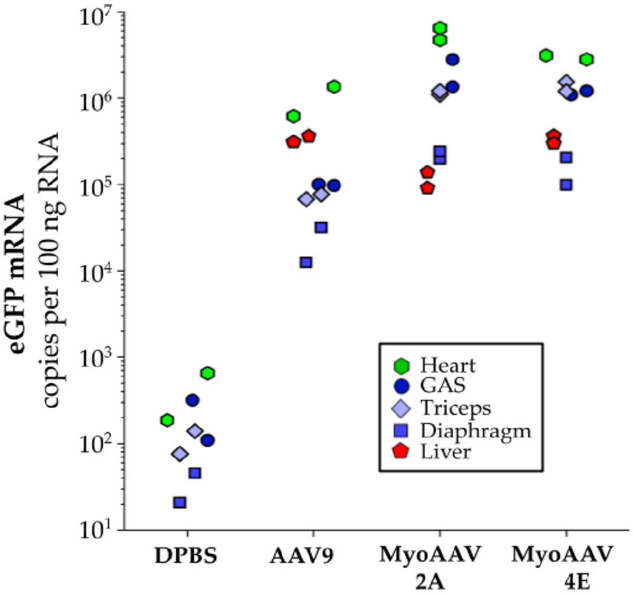

Figure S1. The eGFP expression in the heart and non-target organs following systemic administration of AAV9, MyoAAV 2A, and MyoAAV 4E vectors in adult *mdx* mice ($8×10^{13}$ GC/kg). Four weeks post-injection, tissue samples were collected, followed by RNA extraction and eGFP mRNA quantification by RT-qPCR. See the detailed description in the Materials and Methods section of the manuscript. Each data point represents an organ of a single animal. .

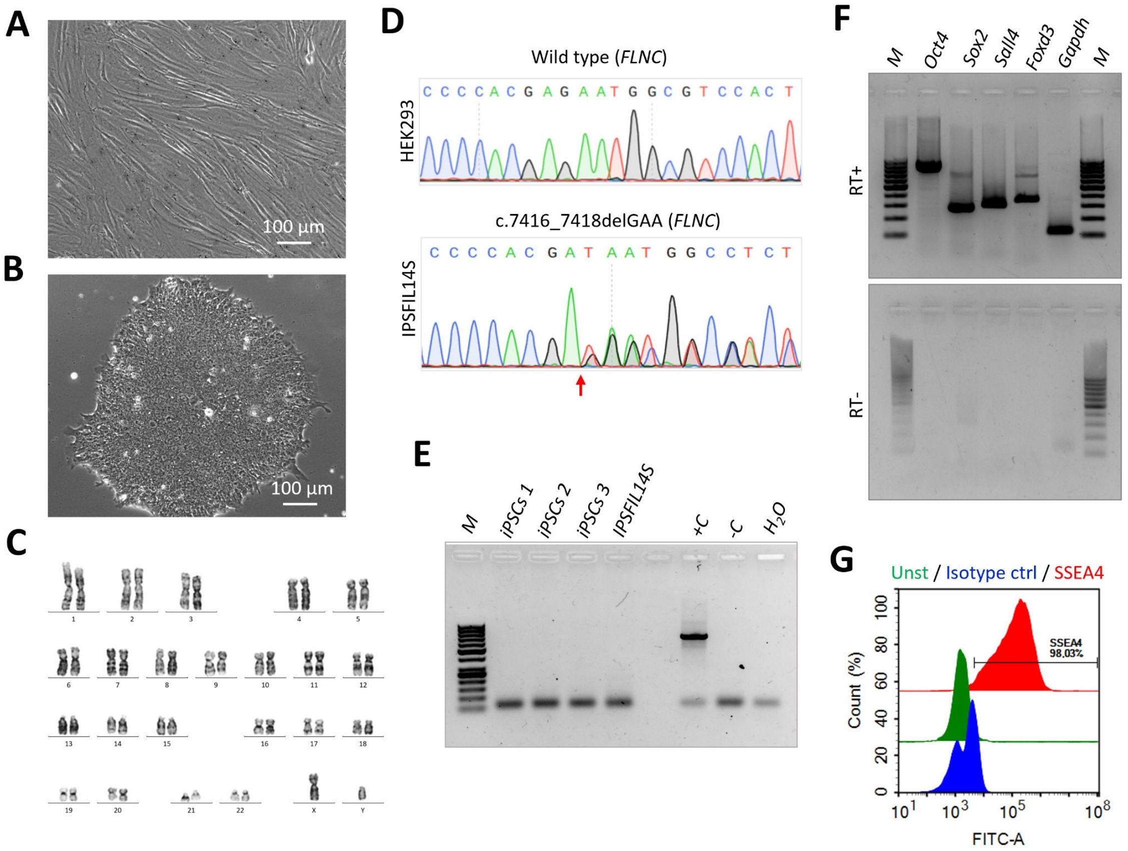

Figure S2. Molecular and genetic characterization of the patient-specific human iPSC line IPSFIL14S. (**a**) A skin biopsy was obtained from a young male patient with a heterozygous deletion. c.7416_7418delGAA in the *FLNC* gene diagnosed with restrictive cardiomyopathy. Primary dermal fibroblasts were used to generate the iPSC clone PSFIL14S. (**b**) The colony of IPSFIL14S cells demonstrates typical morphology for human pluripotent stem cells. (**c**) iPSC line IPSFIL14S has a normal male karyotype (46, XY) and is genetically similar to the primary patient's fibroblasts according to 19 STR and amelogenin locus analyses. (**d**) The Sanger sequencing chromatogram confirms the presence of the mutation in the *FLNC* locus in iPSC line IPSFIL14S; the HEK293 cell line was used as a control. (**e**) PCR analysis verifies the absence of *Mycoplasma spp*. in the cells. M – marker, iPSC 1-3 – human iPSC lines, +K – positive control, and -K – negative control. (**f**) PCR amplicons analyzed by gel electrophoresis demonstrate expression of pluripotency genes *OCT4*, *SOX2*, *SALL4*, and *FOXD3*. RT^+ – cDNA and RT^- – negative control. (**g**) Flow cytometry analysis reveals that 98% of IPSFIL14S cells are SSEA4-positive. Green – negative control (unstained); blue – antibody isotype control; and red – cells stained with the antibodies against SSEA4 (1:50, #MC-813-70, DSHB).

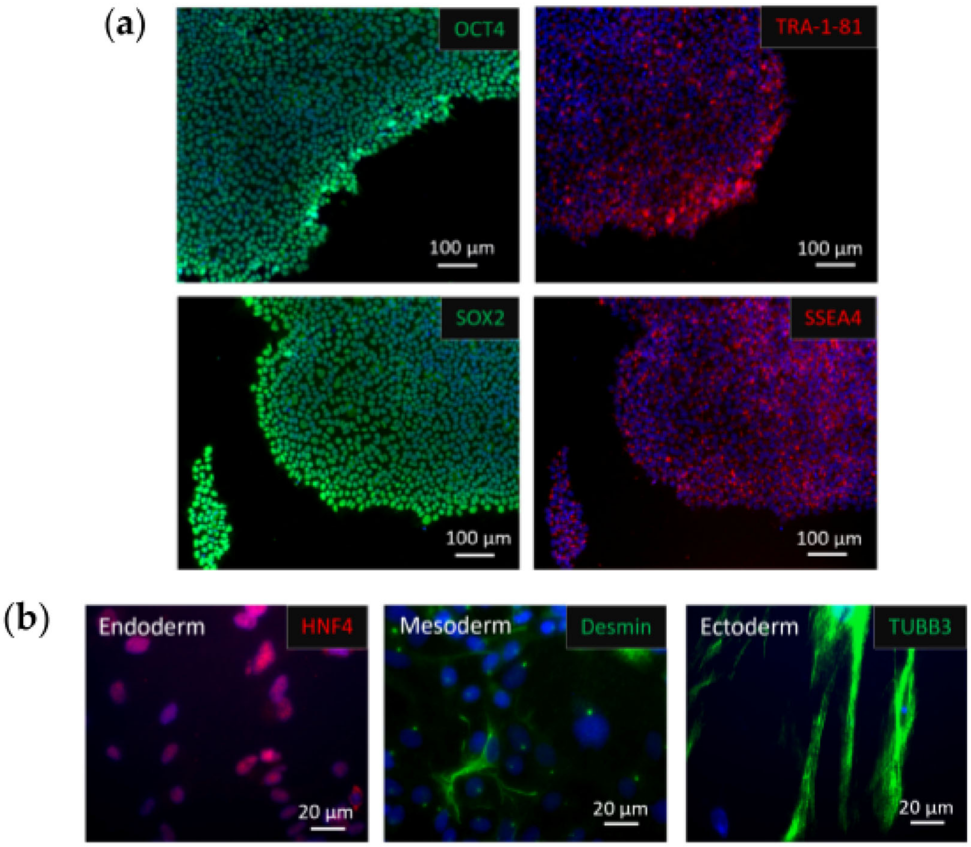

Figure S3. Functional characterization of the patient-specific human iPSC line IPSFIL14S. (**a**) Immunofluorescence microscopy shows positive staining of the IPSFIL14S cells for nuclear (OCT4, SOX2) and surface (TRA-1-81, SSEA4) pluripotency markers. (**b**) Following spontaneous differentiation of the IPSFIL14S cells through the embryoid body stage, the markers of three germ layers were detected in the cultures by immunostaining: entodermal (HNF4), mesodermal (Desmin), and ectodermal (TUBB3).

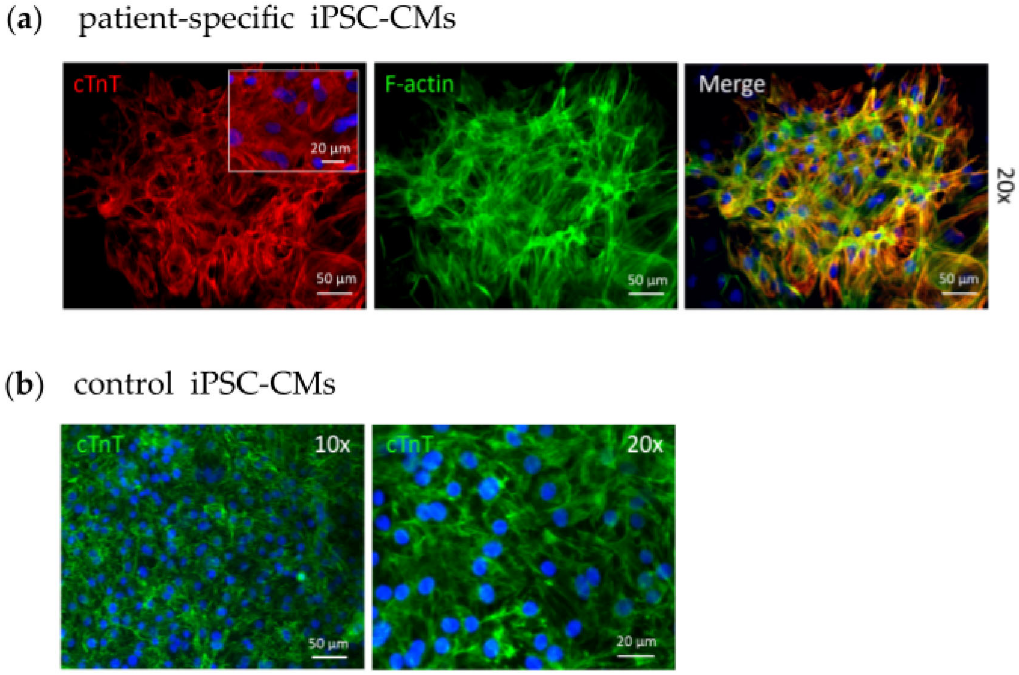

Figure S4. Characterization of human iPSC-derived cardiomyocytes. (**a**) Patient-specific hiPSC-CMs (line IPSFIL14S) were co-stained for the cardiac marker cTnT and cytoskeleton protein F-actin. Nuclei were counterstained with DAPI (blue). Images demonstrate colocalization of cTnT and actin filaments. (**b**) Healthy control hiPSC-CMs (line iPS12) were stained for the cardiac marker cTnT, demonstrating successful cardiac differentiation. For all images, 10x and 20x are magnification factors.

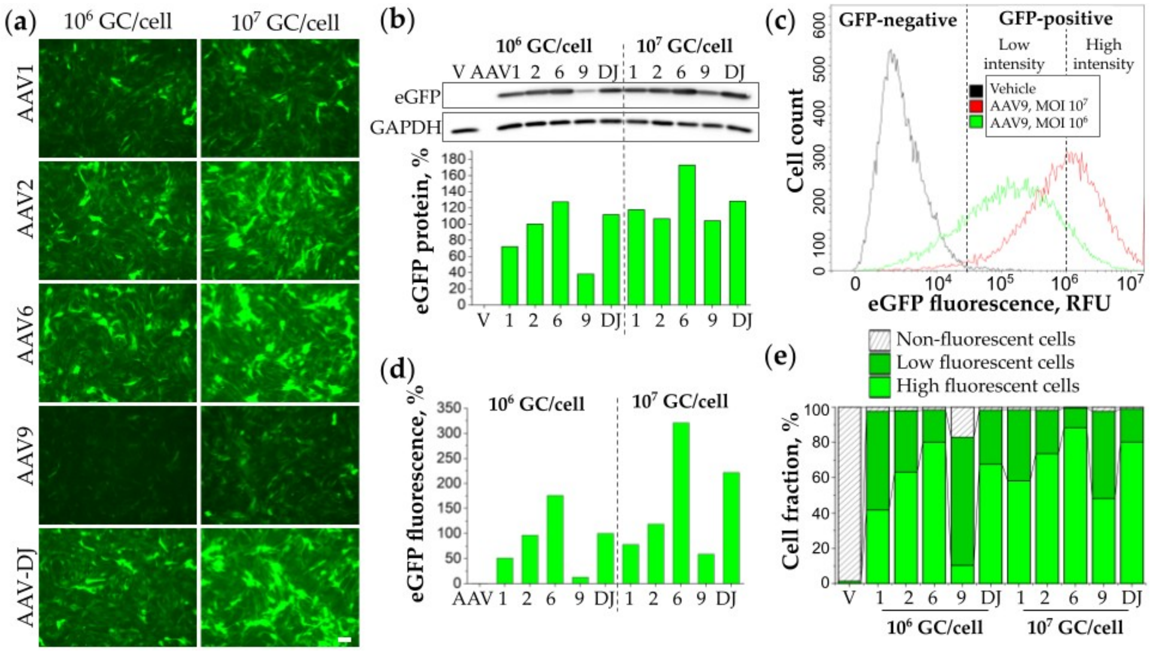

Figure S5. Selecting AAV serotype and multiplicity of infection for efficient transduction of iPSC-derived cardiomyocytes. The control human iPSC line (iPS12) was subjected to differentiation into cardiomyocytes (iPSC-CMs). Cells were transduced with AAV vectors carrying CMV-eGFP expression cassettes and packaged in natural serotypes AAV1, 2, 6, or 9 or chimeric serotype DJ; multiplicity of infection (MOI) roughly corresponded to 10^6 and 10^7 GC per cell. Seven days post-transduc-

tion, eGFP expression was analyzed by fluorescent microscopy, Western blotting, and flow cytometry. (**a**) Representative images of eGFP expression in iPSC-CMs transduced with AAV vectors. Scale bar: 100 µm. (**b**) Western blot (upper panel) and densitometric quantification of the bands (lower bar graph) show the relative level of eGFP protein in cell lysates. (**c**) Flow cytometry was used to determine the % of eGFP-positive cells and intensity of eGFP fluorescence as exemplified using AAV9-transduced iPSC-CMs. Untreated cells were used to set up a threshold for autofluorescence and determine the eGFP-negative cell fraction ("non-fluorescent cells"). The cells with eGFP fluorescence above the threshold were designated eGFP-positive. The fluorescence in the eGFP-positive cell fraction was further subdivided to low and high intensity. (**d**) The relative level of eGFP fluorescence in iPSC-CMs transduced with different AAV serotypes. The average eGFP fluorescence in AAV2-transduced cells at MOI 10^6 GC/ml was set as 100%. (**e**) Fractions of non-fluorescent, low fluorescent, and high fluorescent cells for each AAV serotype, determined as shown in (e). .

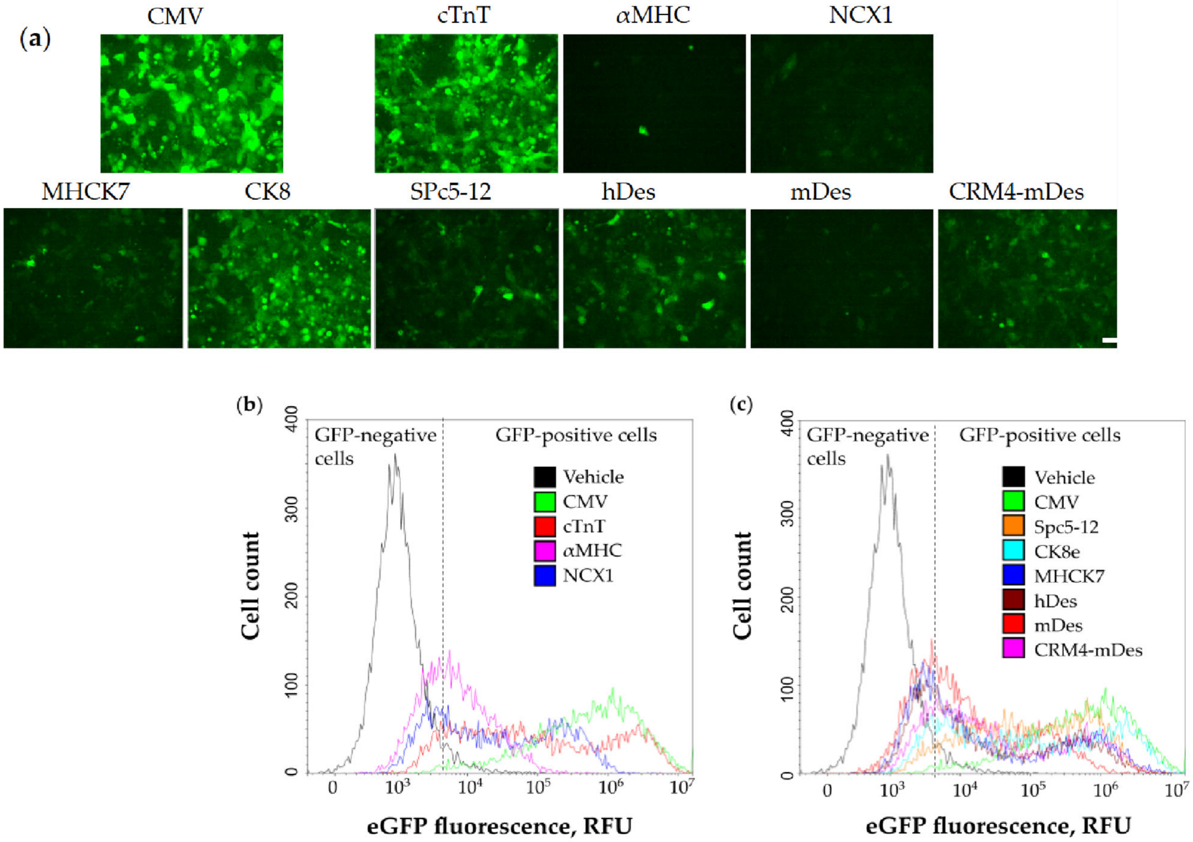

Figure S6. Flow cytometry analysis of promoter-driven eGFP fluorescence in cardiomyocytes *in vitro*. Patient-specific iPSC-CMs (clone IPSFIL14S) were transduced with AAV-DJ vectors encoding the eGFP gene under the control of various promoters at MOI 10^6 of GC/cell. The eGFP fluorescence was analyzed 7 days post-transduction. (**a**) Fluorescent microscopy reveals eGFP expression in AAV-treated cardiomyocytes. Representative images are shown. The flow cytometry was used to quantify relative eGFP fluorescence driven by cardiac (**b**) and pan-muscular (**c**) promoters. Untreated cells were used to set up a threshold for autofluorescence and determine the eGFP-negative cell fraction ("non-fluorescent cells"). The AAV vector with the CMV promoter was used as a positive control.

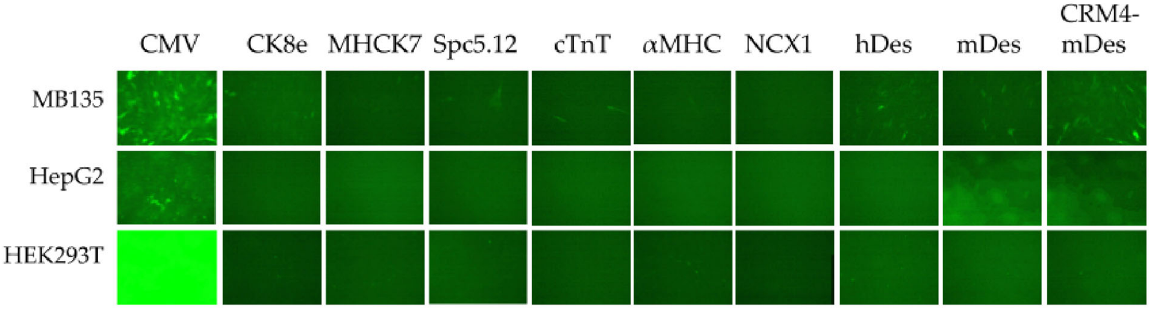

Figure S7. Promoter activity in non-cardiac cell lines. Immortalized cell lines of human myoblasts (MB135), human hepatocarcinoma (HepG2), and human embryonic kidney epithelium (HEK293T) were transduced with AAV-DJ vectors encoding the eGFP gene under the control of cardiac and muscular promoters (MOI of 10^6 GC/cell). A vector with the strong ubiquitous CMV promoter was used as a control. The eGFP expression was detected by fluorescent microscopy 14 days (MB135), 4 days (HepG2), and 3 days (HEK293T) post-transduction. The representative images are shown.

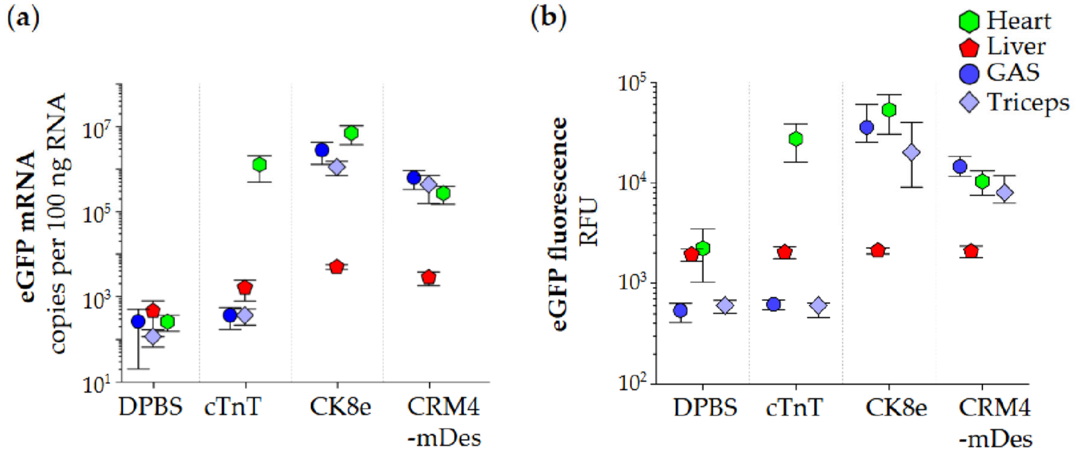

Figure S8. Quantification of eGFP mRNA and fluorescence driven by cardiac and pan-muscular promoters in mice. Eight-week-old C57BL/6J mice were intravenously injected with MyoAAV 2A vectors carrying the eGFP reporter under the control of the cTnT, CK8e, or CRM4-mDesmin promoter (4×10^{13} GC/kg). Four weeks post-injection, eGFP mRNA and fluorescence were quantified in the heart, skeletal muscles, and liver. (**a**) eGFP mRNA was quantified by RT-qPCR as described in the Materials and Methods. (**b**) eGFP fluorescence, which reflects the protein production, was quantified by microplate assay of the tissue lysates. Data presented as mean ± SD, $n = 5$ animals per group.

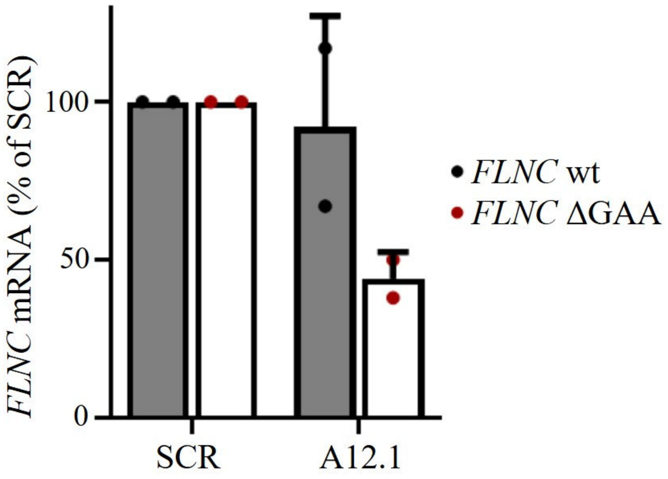

Figure S9. Preliminary data on allele-specific silencing of mutant FLNC by artificial microRNA amiR-A12.1. Human patient-specific iPSC-CMs (derived from clone IPSFIL14S, see Supplementary Materials and Methods) were transduced with AAV-DJ-cTnT-amiR-A12.1 or scrambled control vector AAV-DJ-cTnT-SCR at an MOI of 106. Seven days after treatment, relative levels of wild type (wt) and mutant (ΔGAA) FLNC transcripts were quantified by TaqMan RT-qPCR with allele-specific probes. The transcript levels in the scrambled control samples were set as 100%; n = 2 independent iPSC differentiation and transduction experiments.

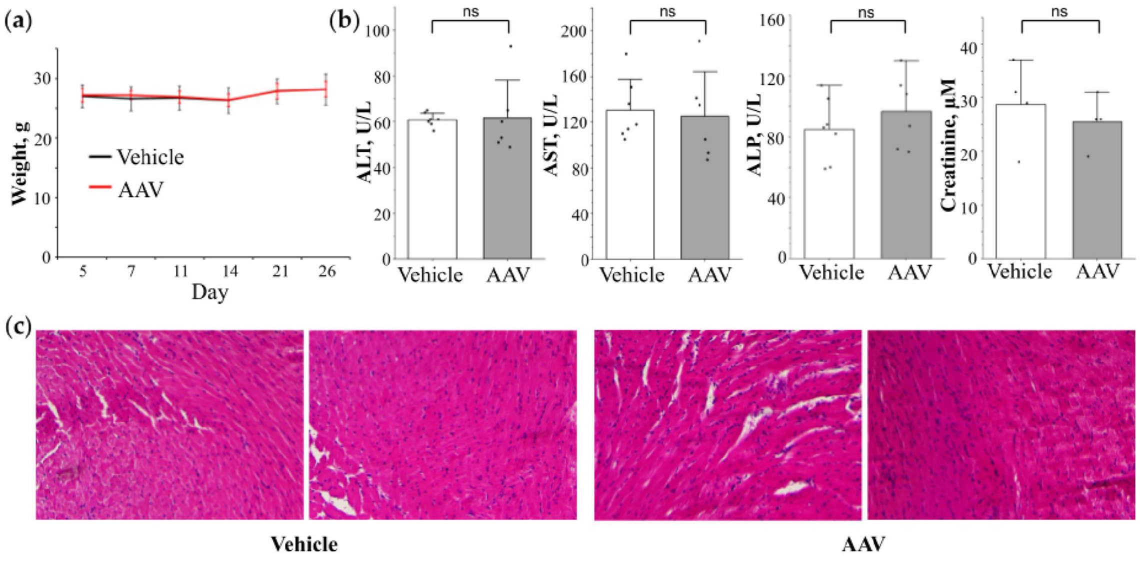

Figure S10. Basic safety study following systemic administration of MyoAAV2A-cTNT-eGFP-amiR-A12.1 into wild-type mice. (a) Body weight dynamics of the AAV-treated mice compared to vehicle-treated control. Data were presented as mean ± SD, n = 7. (b) Levels of alanine transaminase (ALT), aspartate aminotransferase (AST), alkaline phosphatase (ALP), and creatinine in the serum of AAV-treated mice compared to vehicle control. Data were presented as individual data points with mean ± SD. (c) Hematoxylin & eosin staining of heart sections from AAV-treated and control mice. Representative images are shown.

3. Supplementary Table

Table S1. Promoter sequences used in the study.

Promoter (origin)	Size	Description	Coordinates	Sequence, 5’>3’	Ref.
CMV (virus)	508 bp	human cyto-megalovirus immediate-early enhancer and promoter	NC_006273.2; 175571-175064	CGTTACATAACTTACGGTAAATGGCCCGCCTGGCTGACCGCCCAACGACCCCCGCCCATT-<br>GACGTCAATAATGACGTATGTTCCCATAGTAACGCCAATAGGGACTTTCCATTGACGTCAATGG<br>GTGGAGTATTTACGGTAAACTGCCCACTTGGCAGTACATCAAGTGTATCATATGCCAAGTAC-<br>GCCCCCTATTGACGTCAATGACGGTAAATGGCCCGCCTGGCATTATGCCCAGTACATGACCTTA<br>TGGGACTTTCCTACTTGGCAGTACATCTACGTATTAGTCATCGCTATTAC-<br>CATGGTGATGCGGTTTTGGCAGTACATCAATGGGCGTGGATAGCGGTTTGACTCACGGGGATT<br>TCCAAGTCTCCACCCCATTGACGTCAATGGGAGTTTGTTTTGGCACCAAAATCAACGG-<br>GACTTTCCAAAATGTCGTAACAACTCCGCCCCATTGACGCAAATGGGCGGTAGGCGTGTACGG<br>TGGGAGGTCTATATAAGCAGAGCTCGTTTAGTGAACCGTCAGA	
α-MHC (mu-rine)	367 bp	Promoter of myosin heavy chain alpha-isoform gene (*Myh6)*	Chr. 14; NC_000080.7, 55204383 - 55204013	TTGTTCAACTCACCCTTCAGATTAAAAATAACTGAGGTAAGGGCCTGGGTAGGG-<br>GAGGTGGTGTGAGACGCTCCTGTCTCTCCTCTATCTGCCCATCGGCCCTTTGGGGAGGAGGAAT<br>GTGCCCAAGGACTAAAAAAAGGCCATGGAGCCAGAGGGGCGAGGGCAACAGAC-<br>CTTTCATGGGCAAACCTTGGGGCCCTGCTGTCCTCCTGTCACCTCCAGAGCCAAGGGATCAAAG<br>GAGGAGGAGCCAGGACAGGAGGGAAGTGGGAGGGAGGGTCCCAGCAGAG-<br>GACTCCAAATTTAGGCAGCAGGCATATGGGATGGGATATAAAGGGGCTGGAGCACTGAGAGCT<br>GTCAGAGATTTCTCCAACCC	PMID: 1188913
cTnT (chicken)	407 bp	Promoter of cardiac tro-ponin T gene (*TNNT2*)	Chr. 26; NC_052598.1, 811468-811874	GCAGTCTGGGCTTTCACAAGACAGCATCTGGGGCTGCGGCAGAGGGTCGGGTCCGAA-<br>GCGCTGCCTTATCAGCGTCCCCAGCCCTGGGAGGTGACAGCTGGCTGGCTTGTGTCAGCCCCTC<br>GGGCACTCACGTATCTCCGTCCGACGGGTTTAAAATAGCAAAACTCTGAGGCCACACAA-<br>TAGCTTGGGCTTATATGGGCTCCTGTGGGGGAAGGGGGAGCACGGAGGGGGCCGGGGCCGCT<br>GCTGCCAAAATAGCAGCTCACAAGTGTTGCATTCCTCTCTGGGCGCCGGG-<br>CACATTCCTGCTGGCTCTGCCCGCCCCGGGGTGGGCGCCGGGGGGACCTTAAAGCCTCTGCCCC<br>CCAAGGAGCCCTTCCCAGACAGCCGCCGGCACCCACCGCTCCGTGGGACCTAAGCTT	PMID: 14592812
NCX1 (feline)	2004 bp	Promoter of cardiac Na+/Ca2+ transporter gene (*SLC8A1)*	Chr, A3; NC_058370.1; 107190521 - 107192519	CTAGTTAGAAAAAGGCAAGGGAAGAGAGGGCACTGAATGGTCCCTCGG-<br>CACAGCCGCCATCTGCTGGGTCCGCCACTGAGCTACATACGGTCAGCTTTTTAAATACCGTGTCT<br>TAAAAAGTAACCAATTAACTAACTAAATATCATATCTTCATTTCCCTCGGATA-<br>CATTATTTTTATATAGAATATATATTTTTATACAGATAACACATTATTATGCATACGTATAATGTGT<br>AGATACTATACAACATAAAATGCACTAATAAGTGGAATCTCTCTATA-<br>CATTTATGTCAGATTGAAGATATATAGATAATAACATAAAACGTATTACGATATACACTCTACAC<br>ACACACAAACCCACACATATGTCTGATTGAAGGGAGGGAGGGGAAGGTTTGAATGTCATAA-<br>TATTTTGAATGAAAAAAAAAAAATTGCCCCTCCCTGCTCTGGCCTTAGTGCTTTGCACAGAGATTA<br>CTCAAATCCAGAATCCCTCCAACTTGCCATTAGTACAGCTATTTT-<br>GTACCCTCCCCTCTTTTACACAAATTTGAAAATATACGCAAAGGAGAGAGGATAATATAATGAGT<br>CCCCCAGCGACCAGTCAACCCCCCCGCAACAATGATCCTTTTGCCAATCTT-<br>GTGTCATCCATCCTTCCCCTTTTCCTCTGGAAGGCTTTAAGCAACCTTGTTCCATTTCAGTACACAA<br>AAATGAATTATCTCTGCATACCTCGAGTTTATTTTTTCTTCTTCAGATAAGAGAGTC-<br>TATAGGAATTTTAACAGGACTGGGAACAAAAAGGCTAATGGGAAAATGTTATTCGGGAAGTCTC<br>TCACATACGTGCGCGCACGCACACACACACACACGCAC-<br>GCGCGTCACAACAGACTGAAAAAAAGAGAGAAATTTTGAAACAGAGAAGCCAAGTTTTCAGGA<br>ACTGGAAGGTTAAGTGGAGTTTCCACAGCCCCCTGGGGGGAGGACCTCTCACCTA-<br>GAGCTTTATTAGCCGCAGCCAGATTTCTCCTTCCCTACCCTTCAAATTCAGGTCCAGGTTATGCGT<br>CTTTTAAAGTATCGCTGACACTTAATAAATGTAGCAATTAGTCTTCTAG-<br>TCGAATTTACATTCTTTAGCATTATAAAAAAGTTTTCTGAATCTCGAACTGTATGCTAGATGGTAG<br>ATTAGTAGGAATTGCCACAAAGTCCTAAACGAGAAGCTGTGTCCGGAAGAAC-<br>CTGGCCTCTCCTTCTGTCTTGAAGACTTTGATCACTCTGACCTTCCGTTGCTACCTTATCCTTTAAG<br>ACAAATAATTCAGAGTGTTCACAGCTCGTCTTAACTAAA-<br>TAAATACATTAAGTATGTAGGTATTCATTCATGTACAAATTCACATCCATTTGTCCAGACATTCAT<br>TTATACATTCACCATTCACTCAACATTTTGCAGGTCCTGTACATA-<br>TCTTATCGCTGAAATCTTTAAGGATGAAGACCAGACCGTGGTAAGGAGTTATAATTGTGCCTCTG<br>TTTTCATAAGCTAAGCAAACCCACATCCTAAAAGCTCTACGTGGGAATCAGGG-<br>GAAAAGTGATTGTTCCCTAAACGTCCGGGGAGTAGAAATGCCAATCTGAAAGCGTATTTCCAAA<br>AAGGCACAGTGGCAGGACGGCGGGGTGGGAAACGGGCCTGGCTGGAAGGATCTCTATATGTG-<br>TATTTTTATCGTCCATGTGTTGGATGAAGCGGAGAGCTGCCAGATAGCTTCTTTTCCACAGCTTG<br>GAGTTACTGTTGGGAACAGATCCATGTATGGAAGCGAAAGCCGAAAGGCACAGATAAGCAGA-<br>GATCCAGCTATGCAAACCATGTTTAGAGACACTTAAAGGACAAGCATCTCAGGTCCTCTCTTTCT<br>GGTAAATTCGGAGCAGCCATCATACGGGGTCTTTTCTCACATCCAGCCCATGCGGACCGAG-<br>CAGCCCAGACTTGACGGAGGTAGGTGCAGGGCTTTTGTGATGAAACTTATCTAAAGGAGCATTC<br>ATGAATATTCCTTCCTG	PMID: 9111065, 18388932

MHCK7 (murine)	775 bp	Hybrid promoter, conjugate of regulatory region from the muscle creatinine kinase gene (*Mck*) and enhancer from myosin heavy chain alpha-isoform gene (*Myh6*);	Chr14; NC_000080.7; 55204372 - 55204181; Chr7; NC_000073.7 19143764-19143877; Chr14; NC_000073.7 19144664-19145042	CTAGAAGCTGCATGTCTAAGCTAGACCCTTCAGATTAAAAATAACTGAGGTAAGGGCCTGGG-<br>TAGGGGAGGTGGTGTGAGACGCTCCTGTCTCTCCTCTATCTGCCCATCGGCCCTTTGGGGAGGA<br>GGAATGTGCCCAAGGACTAAAAAAAGGCCATGGAGCCAGAGGGGCGAGGGCAACAGAC-<br>CTTTCATGGGCAAACCTTGGGGCCCTGCTGTCTAGCATGCCCCACTACGGGTCTAGGCTGCCCAT<br>GTAAGGAGGCAAGGCCTGGGGACACCCGAGATGCCTGGTTATAATTAACCCAGA-<br>CATGTGGCTGCCCCCCCCCCCCCAACACCTGCTGCCTCTAAAAATAACCCTGTCCCTGGTGGATCC<br>CCTGCATGCGAAGATCTTCGAACAAGGCTGTGGGGGACTGAGGGCAGGCTGTAACAGGCTT-<br>GGGGGCCAGGGCTTATACGTGCCTGGGACTCCCAAAGTATTACTGTTCCATGTTCCCGGCGAAG<br>GGCCAGCTGTCCCCCGCCAGCTAGACTCAGCACTTAGTTTAGGAACCAGTGAG-<br>CAAGTCAGCCCTTGGGGCAGCCCATACAAGGCCATGGGGCTGGGCAAGCTGCACGCCTGGGTC<br>CGGGGTGGGCACGGTGCCCGGGCAACGAGCTGAAA-<br>GCTCATCTGCTCTCAGGGGCCCCTCCCTGGGGACAGCCCCTCCTGGCTAGTCACACCCTGTAGGC<br>TCCTCTATATAACCCAGGGGCACAGGGGCTGCCCTCATTCTACCACCACCTCCACAGCACGAGCT	PMID: 17235310
CK8e (murine)	450 bp	Hybrid promoter based regulatory elements of the muscle creatinine kinase gene (*Mck*)	Chr7; NC_000073.7; 19143779-19143877; Chr7; NC_000073.7; 19144750-19145068	CTAGACTAGCATGCTGCCCATGTAAGGAGGCAAGGCCTGGGGACACCCGA-<br>GATGCCTGGTTATAATTAACCCAGACATGTGGCTGCCCCCCCCCCCCCAACACCTGCTGCCTCTA<br>AAAATAACCCTGCATGCCATGTTCCCGGCGAAGGGCCAGCTGTCCCCCGCCAGCTA-<br>GACTCAGCACTTAGTTTAGGAACCAGTGAGCAAGTCAGCCCTTGGGGCAGCCCATACAAGGCCA<br>TGGGGCTGGGCAAGCTGCACGCCTGGGTCCGGGGTGGGCACGGTGCCCGGGCAAC-<br>GAGCTGAAAGCTCATCTGCTCTCAGGGGCCCCTCCCTGGGGACAGCCCCTCCTGGCTAGTCACA<br>CCCTGTAGGCTCCTCTATATAACCCAGGGGCACAGGGGCTGCCCTCATTCTACCACCAC-<br>CTCCACAGCACAGACAGACACTCAGGAGCCAGCCAGC	PMID: 38970421, 19298131; US10479821B2
SPc5-12 (chicken)	393 bp	Synthetic promoter, combination of conserved SRE, TEF-1, MEF-1, and MEF-2 regulatory elements linked to 144-bp core fragment of the chicken skeletal α-actin promoter		TGCTGCCCATGTAAGGAGGCAAGGCCTGGGGACACCCGA-<br>GATGCCTGGTTATAATTAACCCAGACATGTGGCTGCCCCCCCCCCCCCAACACCTGCTGCCTCTA<br>AAAATAACCCTGCATGCCATGTTCCCGGCGAAGGGCCAGCTGTCCCCCGCCAGCTA-<br>GACTCAGCACTTAGTTTAGGAACCAGTGAGCAAGTCAGCCCTTGGGGCAGCCCATACAAGGCCA<br>TGGGGCTG<br>GCGGCCGCCACCGCGGTGGCGGCCGTCCGCCTTCGGCACCATCCTCACGACACCCAAA-<br>TATGGCGACGGGTGAGGAATGGTGGGGAGTTATTTTTAGAGCGGTGAGGAAGGTGGGCAGGC<br>AGCAGGTGTTGGCGCTCTAAAAATAACTCCCGGGAGTTATTTTTAGAGCGGAGGAATGGTG-<br>GACACCCAAATATGGCGACGGTTCCTCACCCGTCGCCATATTTGGGTGTCCGCCCTCGGCCGGG<br>GCCGCATTCCTGGGGGCCGGGCGGTGCTCCCGCCCGCCTCGA-<br>TAAAAGGCTCCGGGGCCGGCGGCGGCCCACGAGCTACCCGGAGGAGCGGGAGGCGCCAAGCT<br>CTAGAACTAGTGGATCCCCCCAAGTTTGTACAAAAAGCGGCCGC	PMID: 10096290; US10731177B2
hDES (human)	1408 bp	Promoter of desmin gene (*DES*)	Chr. 2; NC_000002.12; 219417037-219418462	ACACACCTACTAGTAACCCCTCCAGCTGGTGATGGCAGGTCTAGGGTAGGACCAG-<br>TGACTGGCICCTAATCGAGCACTCTATTTICAGGGTTTGCATTCCAAAAGGGTCAGGICCAAGAGG<br>GACCTGGAGTGCCAAGTGGAGGTGTAGAGGCACGGCCAGTACCCATGGAGAATGGTG-<br>GATGTCCTTAGGGGTTAGCAAGTGCCGTGTGCTAAGGAGGGGGCTTTGGAGGTTGGGCAGGCC<br>CTCTGTGGGGCTCCATTTTTGTGGGGGTGGGGGCTGGAGCATTATAGGGGGTGG-<br>GAAGTGATTGGGGCTGTCACCCTAGCCTTCCITATCIGACGCCCACCCATGCCTCCTCAGGIACCCC<br>CTGCCCCCCACAGCTCCTCTCCTGTGCCTTGTTTCCCAGCCATGCGTTCTCCTCTATAAA-<br>TACCCGCTCTGGTATTTGGGGTTGGCAGCTGTTGCTGCCAGGGAGATGGTIGGGTTGACATGCG<br>GCTCCTGACAAAACACAAACCCCTGGTGTGTGTGGGCGTGGGTGGTGTGAGTAGGGG-<br>GATGAATCAGGGAGGGGGCGGGGGACCCAGGGGGCAGGAGCCACACAAAGTCIGTGCGGGG<br>GTGGGAGCGCACATAGCAATTGGAAACTGAAAGCTITATCAGACCCTTTCTG-<br>GAAATCAGCCCACTGTTTATAAACTTGAGGCCCCACCCTCGACAGTACCGGGGAGGAAGAGGG<br>CCTGCACTAGTCCAGAGGGAAACTGAGGCTCAGGGCTAGCTCGCCCATAGACATACATGG-<br>CAGGCAGGCTTTGGCCAGGATCCCICCGCCTGCCAGGCGTCTCCCTGCCCTCCCTTCCTGCCTAGA<br>GACCCCCACCCTCAAGCCTGGCTGGTCTTTGCCTGAGACCCAAACCTCTTCGACTTCAAGA-<br>GAATATTTAGGAACAAGGTGGTTTAGGGCCTTTCCTGGGAACAGGCCTTGACCCTTTAAGAAAT<br>GACCCAAAGTCTCTCCTTGACCAAAAAGGGGACCCTCAAACTAAAGGGAA-<br>GCCTCTCTTCTGCTGTCICCCCTGACCCCACICCCCCCCACCCCAGGACGAGGAGATAACCAGGGC<br>TGAAAGAGGCCCGCCTGGGGGCTGCAGACATGCTTGOTGCCTGCCCTGGCGAAGGATTGG-<br>CAGGCTTGCCCGTCACAGGACCOCCGCTGGCIGACTCAGGGGOGCAGGCCTCTTGCGGGGGAG<br>CTGGCCTCCCCGCCCCCACGGCCACGGGCCGCCCITTCCTGGCAGGACAGCGGGATCTT-<br>GCAGCTGTCAGGGGAGGGGAGGCGGGGGCTGATGTCAGGAGGGATACAAATAGTGCCGACG<br>GCTGGGGGCCCTGTCTCCCCTCGCCGCATCCAC-<br>TCTCCGGCCGGOCGCCTGCCCGCCGCCTCCTCCGTGCGCCCGCCAGCCTCGCCCGCGCCGTCACC	PMID: 30700722
mDes (murine)	983 bp	Promoter of desmin (*Des*);	Chr. 1; NC_000067.7; 75336079-75337061	ACCTTGCTTCCTAGCTGGGCCTTTCCTTCTCCTCTATAAATACCAGCTCTGG-<br>TATTTCGCCTTGGCAGCTGTTGCTGCTAGGGAGACGGCTGGCTTGACATGCATCTCCTGACAAAA<br>CACAAACCCGTGGTGTGAGTGGGTGTGGGCGGTGTGAGTAGGGGGATGAATCAGA-<br>GAGGGGGCGAGGGAGACAGGGGCGCAGGAGTCAGGCAAAGGCGATGCGGGGGTGCGACTAC	PMID: 30700722

				ACGCAGTTGGAAACAGTCGTCAGAAGATTCTGGAAACTATCTTGCTGGCTATAAACTT-<br>GAGGGAAGCAGAAGGCCAACATTCCTCCCAAGGGAAACTGAGGCTCAGAGTTAAAACCCAGGT<br>ATCAGTGATATGCATGTGCCCCGGCCAGGGTCACTCTCTGACTAACCGGTAC-<br>CTACCCTACAGGCCTACCTAGAGACTCTTTTGAAAGGATGGTAGAGACCTGTCCGGGCTTTGCCC<br>ACAGTCGTTGGAAACCTCAGCATTTTCTAGGCAACTTGTGCGAATAAAACAC-<br>TTCGGGGGTCCTTCTTGTTCATTCCAATAACCTAAAACCTCTCCTCGGAGAAAATAGGGGGCCTC<br>AAACAAACGAAATTCTCTAGCCCGCTTTCCCCAGGA-<br>TAAGGCAGGCATCCAAATGGAAAAAAAGGGGCCGGCCGGGGGTCTCCTGTCAGCTCCTTGCCC<br>TGTGAAACCCAGCAGGCCTGCCTGTCTTCTGTCCTCTTGGGGCTGTCCAGGGGCG-<br>CAGGCCTCTTGCGGGGGAGCTGGCCTCCCCGCCCCCTCGCCTGTGGCCGCCCTTTTCCTGGCAG<br>GACAGAGGGATCCTGCAGCTGTCAGGGGAGGGGCGCCGGGGGGTGATGTCAGGAGGGC-<br>TACAAATAGTGCAGACAGCTAAGGGGCTCCGTCACCCATCTTCACATCCACTCCAGCCGGCTGCC<br>CGCCCGCTGCCTCCTCTGTGCGTCCGCCCAGCCAGCCTCGTCCACGCCGCCACC	
CRM4-<br>mDes<br>(chimeric)	1424<br>bp	Hybrid pro-<br>moter, conju-<br>gate of murine<br>desmin (*Des*)<br>promoter with<br>CRM4 en-<br>hancer of hu-<br>man myosin<br>light chain<br>(*MYLPF*);	Chr16:<br>NC_000016.10;<br>30372000-<br>30372434<br><br>Chr. 1;<br>NC_000067.7;<br>75336079-<br>7533706	TTCTGAGTCCTCTAAGGTCCCTCACTCCCAACTCAGCCCCATGTCCTGTCAATTCCCAC-<br>TCAGTGTCTGATCTCCTTCTCCTCACCTTTCCCATCTCCCGTTTGACCCAAGCTTCCTGAGCTCTCC<br>TCCCATTCCCCTTTTTGGAGTCCTCCTCCTCTCCCAGAACCCAGTAA-<br>TAAGTGGGCTCCTCCCTGGCCTGGACCCCCGTGGTAACCCTATAAGGCGAGGCAGCTGCTGTCT<br>GAGGCAGGGAGGGGCTGGTGTGGGAGGCTAAGGG-<br>CAGCTGCTAAGTTTAGGGTGGCTCCTTCTCTCTTCTTAGAGACAACAGGTGGCTGGGGCCTCAGT<br>GCCCAGAAAAGAAAATGTCTTAGAGGTATCGGCATGGGCCTGGAGGAGGGGGGACAGGG-<br>CAGGGGGAGGCATCTTCCTCAGGACATCGGGTCCTAGAGGactagtACCTTGCTTCCTAGCTGGG<br>CCTTTCCTTCTCCTCTATAAATACCAGCTCTGGTATTTCGCCTTGGCAGCTGTT-<br>GCTGCTAGGGAGACGGCTGGCTTGACATGCATCTCCTGACAAAACACAAACCCGTGGTGTGAGT<br>GGGTGTGGGCGGTGTGAGTAGGGGGATGAATCAGAGAGGGGGCGAGGGAGACAGGGGCG-<br>CAG-<br>GAGTCAGGCAAAGGCGATGCGGGGGTGCGACTACACGCAGTTGGAAACAGTCGTCAGAAGATT<br>CTGGAAACTATCTTGCTGGCTATAAACTTGAGGGAA-<br>GCAGAAGGCCAACATTCCTCCCAAGGGAAACTGAGGCTCAGAGTTAAAACCCAGGTATCAGTGA<br>TATGCATGTGCCCCGGCCAGGGTCACTCTCTGACTAACCGGTACCTACCCTACAGGCCTAC-<br>CTAGAGACTCTTTTGAAAGGATGGTAGAGACCTGTCCGGGCTTTGCCCACAGTCGTTGGAAACC<br>TCAGCATTTTCTAGGCAACTTGTGCGAATAAAACACTTCGGGGGTCCTTCTT-<br>GTTCATTCCAATAACCTAAAACCTCTCCTCGGAGAAAATAGGGGGCCTCAAACAAACGAAATTCT<br>CTAGCCCGCTTTCCCCAGGA-<br>TAAGGCAGGCATCCAAATGGAAAAAAAGGGGCCGGCCGGGGGTCTCCTGTCAGCTCCTTGCCC<br>TGTGAAACCCAGCAGGCCTGCCTGTCTTCTGTCCTCTTGGGGCTGTCCAGGGGCG-<br>CAGGCCTCTTGCGGGGGAGCTGGCCTCCCCGCCCCCTCGCCTGTGGCCGCCCTTTTCCTGGCAG<br>GACAGAGGGATCCTGCAGCTGTCAGGGGAGGGGCGCCGGGGGGTGATGTCAGGAGGGC-<br>TACAAATAGTGCAGACAGCTAAGGGGCTCCGTCACCCATCTTCACATCCACTCCAGCCGGCTGCC<br>CGCCCGCTGCCTCCTCTGTGCGTCCGCCCAGCCAGCCTCGTCCACGCCGCCACC	PMID:<br>3070072<br>2
